# Bioactive secondary metabolites from endophytic strains of *Neocamarosporium betae* collected from desert plants

**DOI:** 10.3389/fpls.2023.1142212

**Published:** 2023-03-17

**Authors:** Peng Liu, Yue Tan, Jian Yang, Yan-Duo Wang, Qi Li, Bing-Da Sun, Xiao-Ke Xing, Di-An Sun, Sheng-Xiang Yang, Gang Ding

**Affiliations:** ^1^ Key Laboratory of Bioactive Substances and Resources Utilization of Chinese Herbal Medicine, Ministry of Education, Institute of Medicinal Plant Development, Chinese Academy of Medical Sciences and Peking Union Medical College, Beijing, China; ^2^ College of Chemical and Materials Engineering, Zhejiang A&F University, Hangzhou, China; ^3^ State Key Laboratory Breeding Base of Dao-di Herbs, National Resource Center for Chinese Materia Medica, China Academy of Chinese Medical Sciences, Beijing, China; ^4^ Institute of Microbiology, Chinese Academy of Sciences, Beijing, China

**Keywords:** desert plant, endophytic fungus, *Neocamarosporium betae*, polyketides, cytotoxicity, phytotoxicity

## Abstract

Endophytic fungi from desert plants belong to a unique microbial community that has been scarcely investigated chemically and could be a new resource for bioactive natural products. In this study, 13 secondary metabolites (**1–13**) with diverse carbon skeletons, including a novel polyketide (1) with a unique 5,6-dihydro-4*H*,7*H*-2,6-methanopyrano[4,3-*d*][1,3]dioxocin-7-one ring system and three undescribed polyketides (**2**, **7**, and **11**), were obtained from the endophytic fungus *Neocamarosporium betae* isolated from two desert plant species. Different approaches, including HR-ESI-MS, UV spectroscopy, IR spectroscopy, NMR, and CD, were used to determine the planar and absolute configurations of the compounds. The possible biosynthetic pathways were proposed based on the structural characteristics of compounds **1–13**. Compounds **1**, **3**, **4**, and **9** exhibited strong cytotoxicity toward HepG2 cells compared with the positive control. Several metabolites (**2**, **4–5**, **7–9**, and **11–13**) were phytotoxic to foxtail leaves. The results support the hypothesis that endophytic fungi from special environments, such as desert areas, produce novel bioactive secondary metabolites.

## 1 Introduction

**Figure 1:**
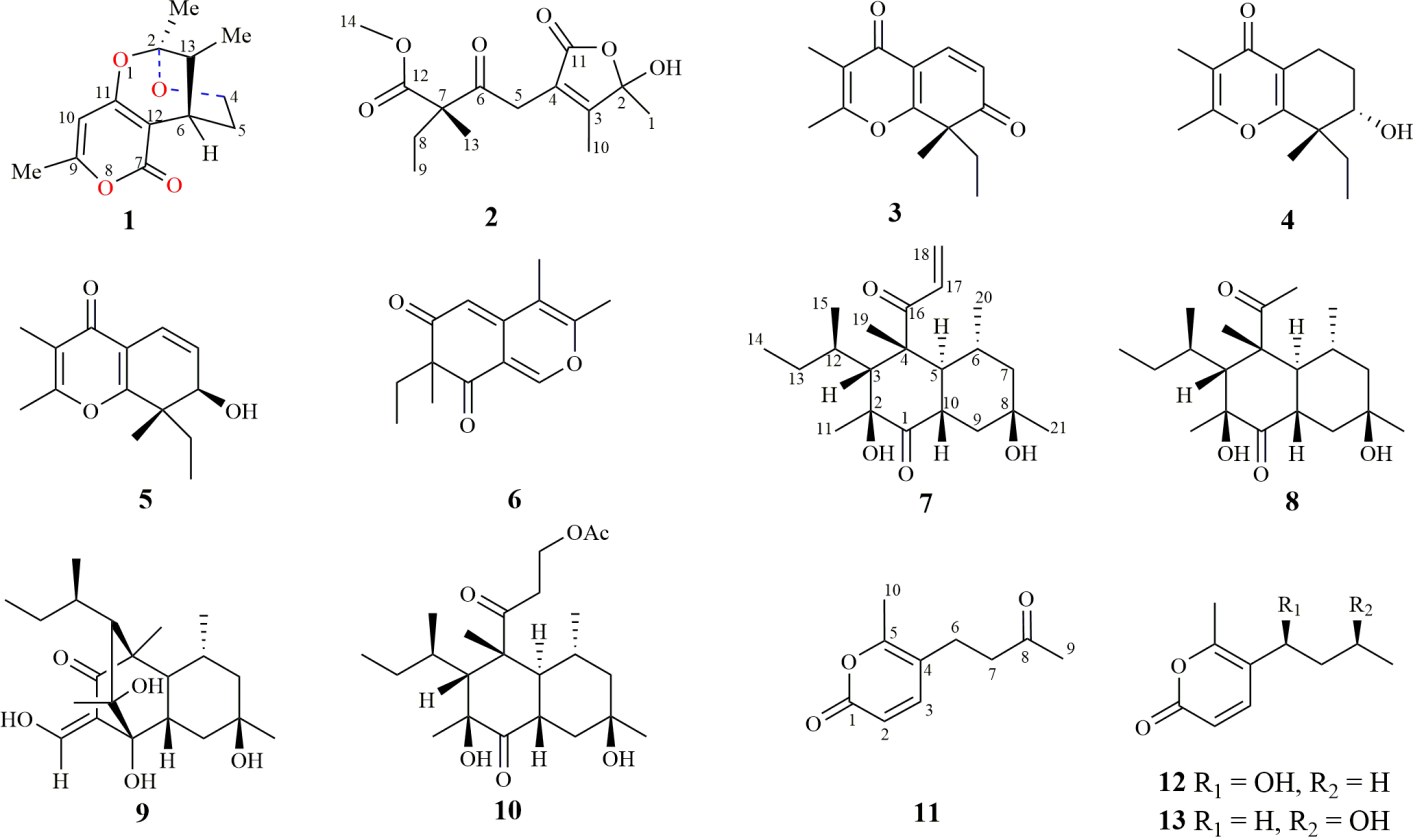
Chemical structures of compounds 1–13.

Plant endophytic fungi are one of the significant sources of novel bioactive compounds (Tan and Zou, 2001; Zhang et al., 2006; Kharwar et al., 2011; Yang et al., 2012; Adnani et al., 2017). and can produce bioactive drug molecules, such as the anticancer compound taxol (Kharwar et al., 2011). Furthermore, *Alternaria oxytropis* can biosynthesize the neurological toxin swainsonine to protect its host plant against livestock (Braun et al., 2003; Cook et al., 2011; Cook et al., 2013; Grum et al., 2013; Cook et al., 2014). Large-scale chemical investigations during the last two decades have lowered the chance of obtaining novel bioactive compounds from plant endophytic fungi inhabiting common environments. Therefore, fungi from special biotopes have become hot spots for isolating new natural products (Tian et al., 2017; Zhang et al., 2018a). Compared with plants in common environments, desert plants must develop strategies to adapt to extreme conditions, such as strong ultraviolet radiation, high-concentration saline-alkali stress, drought, and large temperature fluctuations. Endophytic fungi inhabiting desert plants can produce secondary metabolites, with different biological or ecological functions, to help the host plants tolerate extreme conditions during their mutualistic symbiotic relationship. Thus, endophytic fungi inhabiting desert plants belong to a unique fungal community that can biosynthesize novel bioactive secondary metabolites. Recently, our group chemically investigated fungi collected from desert plants across Northwest China and isolated a series of small molecules with novel skeletons and various biological activities (Zhang et al., 2018b; Song et al., 2019; Tan et al., 2019; Li et al., 2020; Zhang et al., 2021; Tan et al., 2022; Xu et al., 2022). Thirteen secondary metabolites (**1–13**) (**Figure 1**), including four undescribed structures (**1**, **2**, **7**, and **11**), with different biosynthetic origins were obtained from the endophytic fungus *Neocamarosporium betae* (synonym: *Phoma betae*), isolated from *Suaeda glauca* Bunge (Chenopodiaceae) and *Nitraria roborowskii* Kom. (Zygophyllaceae). This study describes the extraction and purification, structural characterization, biosynthesis, and biological evaluation of these compounds.

## 2 Materials and methods

### 2.1 Instruments and equipment

HR-ESI-MS data, circular dichroism (CD) spectra, infrared (IR) spectra, ultraviolet and visible (UV–vis) spectra, and optical rotations were examined using the instrumentation described (Tan et al., 2022). Nuclear magnetic resonance (NMR) spectroscopy was performed on a 500 Bruker spectrometer (^1^H: 500 MHz, ^13^C: 125 MHz) and a 600 Bruker spectrometer (^1^H: 600 MHz, ^13^C: 150 MHz). Semipreparative HPLC isolation was performed by using the LC-52 equipment [Separation (Beijing) Technology Ltd., China] equipped with a column of H&E-SP ODS-A (5 μm, 250 × 10 mm). Sephadex LH-20 was purchased from Pharmacia (Biotech, Sweden).

### 2.2 Strain and fermentation

*Neocamarosporium betae* (CGMCC3.19915; GenBank Accession: ON945545) isolated from *S. glauca* Bunge and *N. betae* (CGMCC3.20844; GenBank Accession: ON945546) isolated from *N. roborowskii* Kom. were identified by Dr. Bing-da Sun. Endophytic fungi were cultured on PDA at 25°C for 1 week. Fresh mycelia were incubated in a solid medium (80 ml of distilled water and 60.0 g of rice in 500 ml flasks) to be fermented at room temperature for 4 weeks.

### 2.3 Extraction and isolation

Ferments of *N. betae* (CGMCC3.19915) isolated from *S. glauca* (Bunge) were extracted three times with ethyl acetate. The extract was evaporated *in vacuo* until dry, affording 19.8 g. Then, nine fractions (Fr.1–Fr.9) were derived from the crude extract by stepwise fractionation using PE–AC (100:0, 100:1, 80:1, 60:1, 40:1, 20:1, 10:1, 5:1, and 0:1). Using a silica gel column, Fr.3 (1.9 g) was fractionated for betaethrone (**1**; 1.8 mg, *t*_R_ = 18.1 min) as well as spiciferone A (**3**; 6.1 mg, *t*_R_ = 19.6 min) through semipreparative RP-HPLC (55% acetonitrile in H_2_O). Fr.5 (610.8 mg) yielded spiciferinone (**6**; 1.0 mg, *t*_R_ = 24 min) through semipreparative RP-HPLC (60% methanol in H_2_O). Separation from Fr.6 (836.1 mg) through gel column chromatography (CH_2_Cl_2_–MeOH, 1:1), followed by purification with semipreparative RP-HPLC (55% methanol in H_2_O), yielded dehydrospicifernin (**2**; 1.7 mg, *t*_R_ = 20.4 min). Similarly, Fr.7 (530.8 mg) was separated with gel column chromatography (CH_2_Cl_2_–MeOH, 1:1) and semipreparative RP-HPLC (55% methanol in H_2_O) to yield compound **4** (1.1 mg, *t*_R_ = 38.7 min).

Ferments of *N. betae* isolated from *N. roborowskii* Kom. were extracted three times with ethyl acetate. A total of 43.8 g of crude extract was obtained by evaporation of solvent to dryness in vacuum. Then, eight fractions (Fr.1–Fr.8) were separated from the extract progressively by using silica column chromatography, eluting with PE–AC (100:0–0:1). Fr.4 (1.9 g) was isolated with gel column chromatography (acetone), affording three subfractions (Fr.4.1–Fr.4.3). Eight subfractions (Fr.4.2.1–Fr.4.2.8) were separated from Fr.4.2 (1.3 g) using an ODS C-18 column and methanol–H_2_O (2:3–1:0). Betaenone E (**8**; 9.3 mg, *t*_R_ = 45.2 min) was purified from Fr4.2.2 (23.7 mg) through semipreparative HPLC (45% CH_3_CN in H_2_O). Betaenone A (**9**; 7 mg, *t*_R_ = 36.7 min) was purified from Fr.4.2.3 (47 mg) through semipreparative HPLC (49% CH_3_CN in H_2_O). Betaeniol (**7**; 5.1 mg, *t*_R_ = 30.7 min) was obtained from Fr.4.2.4 (24.6 mg) through semipreparative HPLC (60% CH_3_CN in H_2_O). Seven subfractions (Fr.5.1–Fr.5.7) were isolated from Fr.5 (2.62 g) by using a silica gel column (100–200 mesh) and a gradient of PE–AC (50:1–0:1). Fr.5.4 (577.7 mg) was then isolated using gel column chromatography (CH_2_Cl_2_–MeOH, 1:1) to afford six subfractions (Fr.5.4.1–Fr.5.4.6). The purification of betaenone F (**10**; 6.9 mg, *t*_R_ = 29.6 min) from Fr.5.4.4 (155.3 mg) was performed using semipreparative RP-HPLC (MeOH/H_2_O, 60% for 5 min, then 60%−100% for 30 min). Spiciferol A (**5**; 5.6 mg, *t*_R_ = 28.4 min) and betalactone (**11**; 4.3 mg, *t*_R_ = 10 min) were purified from Fr.5.4.5 (123.0 mg) using semipreparative RP-HPLC (MeOH/H_2_O, 55% for 5 min, then 55%−60% for 20 min). Xylariolide D (**12**; 3.2 mg, *t*_R_ = 13.5 min) was purified from Fr.5.4.6 (49.6 mg) through semipreparative RP-HPLC (MeOH/H_2_O, 60% for 5 min, then 60%–89% for 22 min). Fr.5.5 (1.4 g) was isolated using a silica gel column with dichloromethane–acetone (100:0–0:1), and then semipreparative RP-HPLC (MeOH/H_2_O, 55% for 5 min, then 55%–70% for 15 min) treatment was used to obtain xylariolide F (**13**; 8.4 mg, *t*_R_ = 13.6 min).

*Betaethrone (**1**)*: White powder; [*α*]**25**_D_ +8 (*c* 0.1, MeOH), UV (MeOH) *λ*_max_ (log *ϵ*) 215 (3.19), 282 (2.54) nm; CD (*c* 0.57 mM, MeOH): *λ*(Δ*ϵ*) 212 (−0.48); IR (neat) *n*_max_ 3,378, 2,944, 2,844, 1,719, 1,599, 1,494, 1,364, 1,266, 1,154, 1,027, and 837 cm^−1^; for ^1^H-NMR and ^13^C-NMR data, see **Table 1**; (+)-HR-ESI-MS: *m*/*z* 237.1122 [M+H]^+^ (calcd. for C_13_H_17_O_4_, 237.1127).

**Table 1:** ^1^H- (500 MHz) and ^13^C-NMR (125 MHz) data for compounds **1** and **2** in CDCl_3_.

No.	1		2	
	*δ*_C_, type	*δ*_H_ (*J* in Hz)	*δ*_C_, type	*δ*_H_ (*J* in Hz)
1			23.2, CH_3_	1.65, d (1.5)
2	103.3, C		105.7, C	
3			163.1, C	
4	60.2, CH_2_	3.80, dd (12.0, 6.0) 3.58, td (12.0, 3.5)	121.7, C	
5	21.7, CH_2_	2.16, tdd (12.0, 6.0, 3.5) 1.34, m	33.0, CH_2_	3.45, m
6	28.8, CH	2.98, dd (7.0, 3.5)	204.0, C	
7	163.6, C		60.1, C	
8			28.0, CH_2_	2.01, m 1.89, m
9	161.0, C		8.6, CH_3_	0.87, t (7.5)
10	99.3, CH	5.83, s	11.0, CH_3_	1.97, d (1.5)
11	102.0, C		170.4, C	
12	166.0, C		173.1, C	
13	34.9, CH	1.77, qd (7.0, 3.0)	18.6, CH_3_	1.40, d (10.0)
14			52.6, CH_3_	3.77, s
2-CH_3_	25.2	1.44, s		
9-CH_3_	19.9	2.21, s		
13-CH_3_	13.6	1.25, d (7.0)		

*Dehydrospicifernin (**2**)*: Yellow oil; [*α*]**25**_D_ +5 (*c* 0.1, MeOH); UV (MeOH) *λ*_max_ (log *ϵ*) 208 (3.74); IR (neat) *n*_max_ 3,390, 2,925, 2,872, 1,689, 1,616, 1,455, 1,377, 1,313, 1,116, 1,032, and 895 cm^−1^; for ^1^H-NMR and ^13^C-NMR data, see **Table 1**; (+)-HR-ESI-MS: *m*/*z* 285.1329 [M+H]^+^ (calcd. for C_14_H_21_O_6_, 285.1338).

*Betaeniol (**7**)*: Crystal powder; [*α*]**25**_D_ +29 (*c* 0.1, MeOH); UV (MeOH) *λ*_max_ (log *ϵ*) 218 (3.69); CD (*c* 0.39 mM, MeOH): *λ*(Δ*ϵ*) 203 (−3.92), 237 (0.11), 296 (1.42); IR (neat) *n*_max_ 3,480, 2,963, 2,877, 1,704, 1,682, 1,603, 1,455, 1,396, 1,378, 1,250, 1,172, 1,136, 1,030, 1,008, and 923 cm^−1^; for ^1^H-NMR and ^13^C-NMR data, see **Table 2**; (+)-HR-ESI-MS: *m*/*z* 351.2531 [M+H]^+^ (calcd. for C_21_H_35_O_4_, 351.2535).

**Table 2:** ^1^H- (600 MHz) and ^13^C-NMR (150 MHz) data for compounds **7** and **11** in CDCl_3_.

No.	7		11	
	*δ*_C_, type	*δ*_H_ (*J* in Hz)	*δ*_C_, type	*δ*_H_ (*J* in Hz)
1	217.3, C		162.5, C	
2	77.6, C		113.6, CH	6.14, d (9.6)
3	57.5, CH	1.53, s	146.9, CH	7.19, d (9.6)
4	51.3, C		114.1, C	
5	46.1, CH	2.58, dd (13.2, 9.6)	159.1, C	
6	29.3, CH	1.78, m	23.2, CH_2_	2.56, t (7.2)
7	47.5, CH_2_	1.15, m 1.56, m	43.0, CH_2_	2.64, t (7.2)
8	69.9, C		206.8, C	
9	41.8, CH_2_	2.31, dd (13.8, 3.6) 1.31, dd (13.8, 11.4)	30.1, CH_3_	2.16, s
10	40.6, CH	2.63, ddd (13.2 11.4, 3.6)	17.3, CH_3_	2.25, s
11	23.8, CH_3_	1.60, s		
12	35.4, CH	1.78, m		
13	25.1, CH_2_	1.38, m 1.46, m		
14	13.4, CH_3_	0.74, t (7.2)		
15	23.5, CH_3_	1.11, d (7.2)		
16	203.3, C			
17	132.6, CH	6.99, dd (16.8, 10.2)		
18	128.3, CH_2_	6.37, dd (16.8, 1.2) 5.71, dd (10.2, 1.2)		
19	20.8, CH_3_	1.42, s		
20	22.3, CH_3_	0.60, d (6.6)		
21	31.3, CH_3_	1.26, s		

*Betalactone (**11**)*: Colorless oil; UV (MeOH) *λ*_max_ (log *ϵ*) 205 (3.43), 307 (3.35); IR (neat) *n*_max_ 3,502, 2,935, 1,715, 1,637, 1,555, 1,361, 1,165, 1,086, and 824 cm^−1^; for ^1^H-NMR and ^13^C-NMR data, see **Table 2**; (+)-HR-ESI-MS: *m*/*z* 181.0853 [M+H]^+^ (calcd. for C_10_H_13_O_3_, 181.0865).

### 2.4 Cytotoxic evaluation using the MTT assay

The cytotoxicity of compounds **1–13** was tested against three cancer cell lines, namely, MCF7, B16, and HepG2, using the MTT assay. *cis*-Platinum was used as a positive control. Cytotoxic assessment was as described (Wang et al., 2020).

### 2.5 Phytotoxicity assay

The phytotoxicity of different concentrations of the pure compounds was tested on the leaves of green foxtail and corn using a leaf puncture test (Li et al., 2020). Fresh leaves were cut into 4-cm rectangles using scissors, washed three times with 75% alcohol, and dried in a sterile glass Petri dish, and sterile wet filter paper was placed on the bottom. Next, 5-mm diameter filter paper discs, prepared using a hole punch, were disinfected for 30 min at 121°C. The compounds were prepared as 1 mg/ml solutions with dimethyl sulfoxide. On each leaf segment, three benign lesions were made equidistant from each other using forceps. A disc was placed over the lesion, and 20 μl of dimethyl sulfoxide or test solution was added to each disc. The experiment was repeated three times and the results were recorded after 72 h.

## 3 Results and discussion

**Figure 2:**
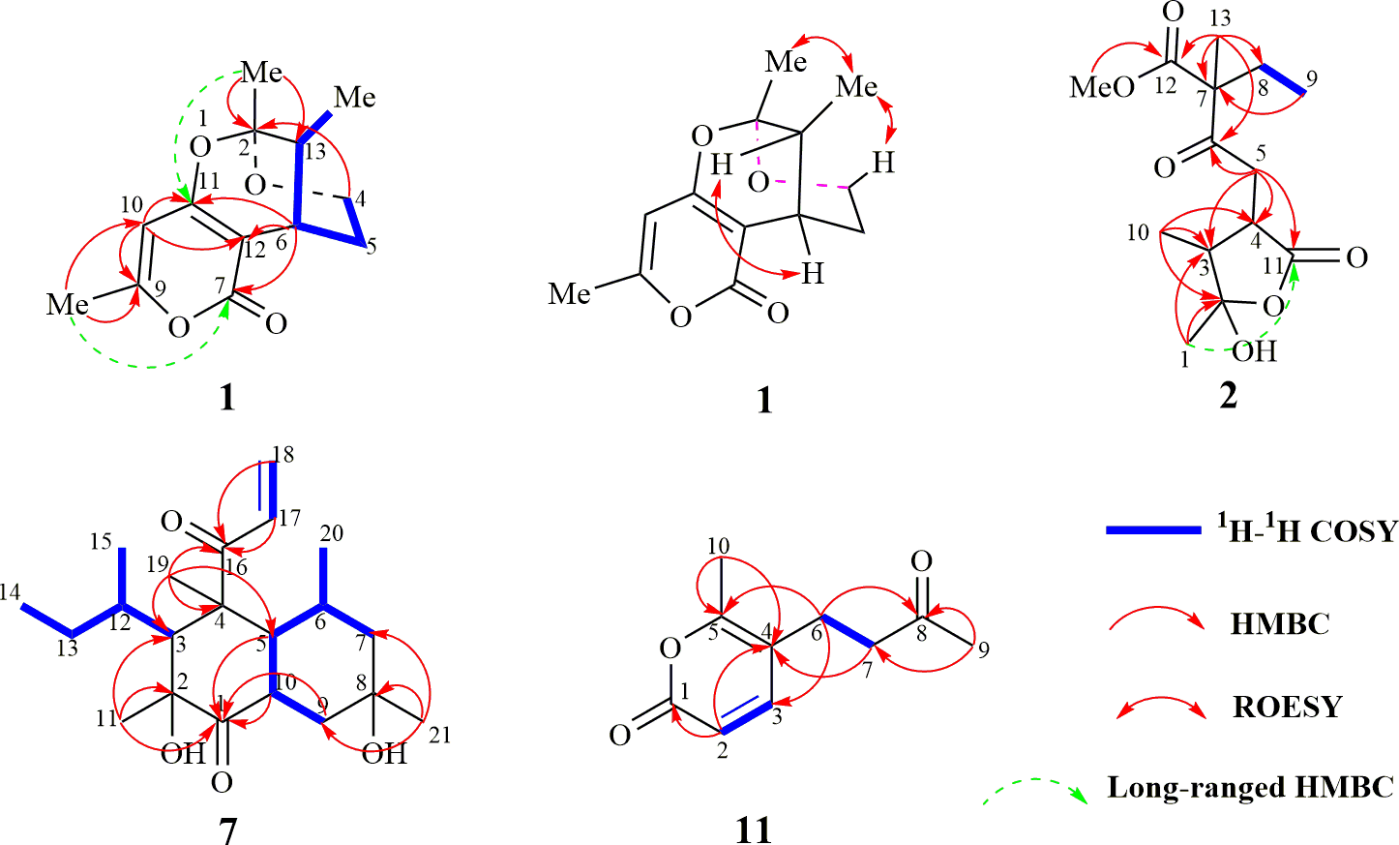
Key 2D-NMR correlations of compounds 1, 2, 7, and 11.

**Figure 3:**
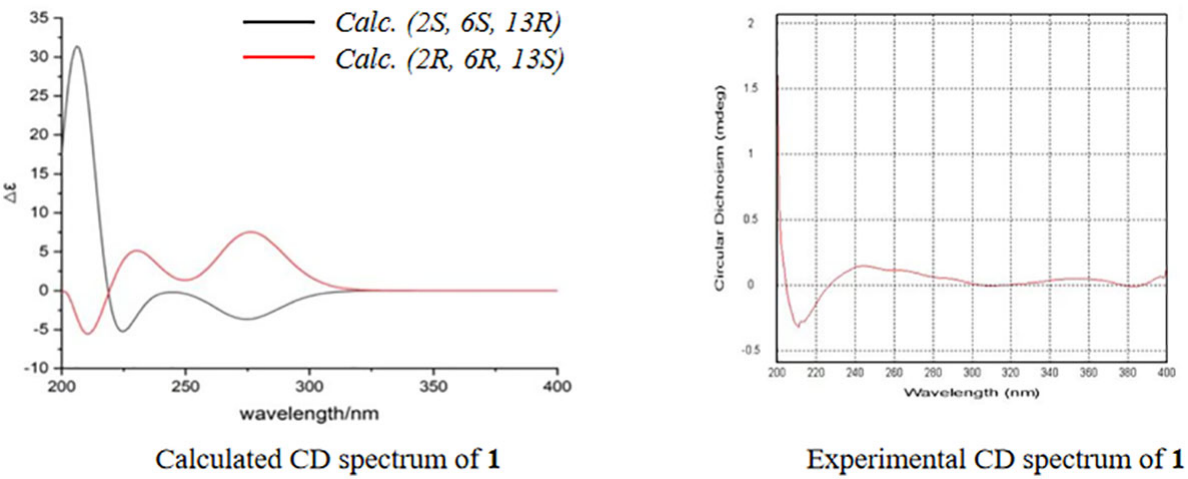
Calculated and experimental circular dichroism spectrum of compound **1**.

The molecular formula of **1** was determined as C_13_H_16_O_4_ using HR-ESI-MS (*m*/*z* 237.1122 [M+H]^+^; calcd. 237.1127), with six degrees of unsaturation. Three methyl, two sp^3^ methylene (one oxygenated), two sp^3^ methine, one quaternary carbon, four olefinic carbon, and one carbonyl group were suggested to be present in the structure of **1** (**Table 1**) based on the analysis of ^1^H, ^13^C, and HMBC NMR spectra. These data accounted for all resonances of ^1^H- and ^13^C-NMR spectra, with three rings, in structure **1**. The ^1^H–^1^H COSY correlations gave an isolated proton spin system corresponding to -CH_2_-4/CH_2_-5/C-6/C-13/13-Me. Complete connectivity was established through HMBC spectrum analysis (**Figure 2**). The 9-Me/C-9/C-10/C-11/C-12 fragment was constructed based on the HMBC correlations of 9-Me to C-9 and C-10 and of H-10 to C-9, C-11, and C-12; C-12 was connected to C-6, C-7, and C-11 by correlations from H-6 to C-7 (*δ*_C_ = 163.6), C-11, and C-12. The weak, long-range HMBC correlation from 9-Me to C-7 confirmed a 2*H*-pyran-2,4(*3H*)dione ring (A ring) in the structure of **1** (Ding et al., 2012). C-2 was linked to C-13 and 2-Me due to the HMBC correlations of 2-Me with C-2 and C-13, and an ether bond was formed between C-2 and C-4 according to the HMBC correlation of H-4 with C-2. Considering the chemical shift values of C-2 (*δ*_C_ = 103.3)/C-11 (*δ*_C_ = 166.3) and degrees of unsaturation, an ether bond might be present between C-2 and C-11. This hypothesis was supported by the weak long-range HMBC correlation from 2-Me to C-11. Thus, the planar structure of 1 was established as possessing a unique 5,6-dihydro-4*H*,7*H*-2,6-methanopyrano[4,3-*d*][1,3]dioxocin-7-one skeleton, which was the first report in nature. The relative configuration of **1** was established based on its ROESY correlations (**Figure 2**), whereas the stereochemistry of **1** was resolved by CD spectroscopy. The ECD spectrum of **1** resembled the calculated ECD curve of (2*S*, 6*S*, 13*R*)-**1**, but was opposite to that of (2*R*, 6*R*, 13*S*)-**1** (**Figure 3**), which established the absolute configuration of **1** (**Figure 1**).

The molecular formula of compound **2** was determined to be C_14_H_20_O_6_ according to the HR-ESI-MS data (*m*/*z* 285.1329 [M+H]^+^; calcd. 285.1338). The structure of **2** was mainly elucidated using 2D-NMR spectral correlations. The ^1^H–^1^H COSY correlations of **2** showed an isolated proton spin system, corresponding to -C-8/C-9-, and the remaining connection was established through the HMBC spectrum. The quaternary carbon (C-7) was connected with C-6, C-8, C-12, and C-13, based on correlations from 13-Me to C-6, C-7, C-8, and C-12. The connection of C-4 with C-3, C-5, and C-11 was achieved through the HMBC correlations of -CH_2_-5 with C-3, C-4, and C-11. HMBC correlations from 1-Me/10-Me to C-2 and C-3 led to the connection of -C-1/C-2/C-3/C-10-. C-5 was connected to the keto-group C-6, whereas 14-OMe was anchored with C-12, according to the correlations from -CH_2_-5 to C-6/C-7 and 14-OMe to C-12, respectively. The chemical shift values and degrees of unsaturation of C-2/C-11, together with the weak long-range HMBC correlation from 1-Me to C-11, indicated the presence of a γ-hydroxyl-γ-lactone unit in **2**, possessing the same unique carbon skeleton found in spicifernin (Nakajima et al., 1990). Spicifernin existed as an equilibrium mixture of keto acid and a γ-hydroxyl-γ-lactone in a solvent (Nakajima et al., 1990). A set of double carbon signals was observed in the ^13^C-NMR spectrum of **2**, suggesting that a pair of stereoisomers might be present in **2** due to the hemiacetal hydroxyl at C-2 (**Table 1**).

The molecular formula of compound **7** was determined to be C_21_H_34_O_4_ according to the HR-ESI-MS spectra (*m*/*z* 351.2531, [M+H]^+^; calcd. 351.2535), with five degrees of unsaturation. Comparison with the NMR spectra of **8–10** indicated that compound **7** possessed the same carbon skeleton as compounds **8–10**, except that an additional terminal double bond (*δ*_H-17_ = 6.99, *J* = 16.8 Hz, 10.2 Hz; *δ*_H-18a_ = 6.37, *J* = 16.8 Hz, 1.2 Hz; *δ*_H-18b_ = 5.71, *J* = 10.2 Hz, 1.2 Hz) was observed in the NMR spectra (**Table 2**) (Ichihara et al., 1983a; Ichihara et al., 1983b). The ^13^C-NMR chemical shift value of C-16 in compound **7** was *δ*_C_ = 203.3, which was smaller than that for compounds **8** and **10** (Ichihara et al., 1983a; Ichihara et al., 1983b). These findings suggest that the additional terminal olefins were connected with C-16 to form an α, β-unsaturated keto-group, causing the ^13^C-NMR chemical shift value to be high-fielded. This hypothesis was further confirmed by the HMBC correlations from CH_2_-18/H-17 to C-16. The ^1^H–^1^H COSY correlations together with coupling constant analysis determined the connectivity of H-17/CH_2_-18 (**Figure 2**). The absolute configuration of compound **7** was the same as that of compound **10**, considering their similar CD spectra (**Figure S23**, **Supplementary data**).

The molecular formula of compound **11** was determined to be C_10_H_12_O_3_ according to the HR-ESI-MS (*m*/*z* 181.0853, [M+H]^+^; calcd. 181.0865) and NMR data (**Table 2**), with five degrees of unsaturation. The ^1^H- and ^13^C-NMR spectra of compound **11** displayed similar resonances as those of compound **13**, except that 9-Me in **11** was a singlet peak compared with the doublet (*δ*_9-Me_ = 2.25) in **13**. An additional keto-group signal (*δ*_C_ = 206.8) was observed in the ^13^C-NMR spectrum of **13**. These resonance differences in compounds **11** and **13** implied that the hydroxyl at C-8 in 13 was transformed into the corresponding keto group in **11**. This hypothesis was further supported by the HMBC correlations of 9-Me with C-7 and C-8 (**Figure 2**), which established the structure of **11**.

Compounds 3–6, 8–10, and 12, 13 were determined to be spiciferone A (**3**) (Tan et al., 2018), (7*S*,8*S*)-8-ethyl-7-hydroxy-2,3,8-trimethyl-5,6,7,8-tetrahydro-4H-chromen-4-one (**4**) (Tan et al., 2018), spiciferol A (**5**) (Edrada et al., 2000), spiciferinone (**6**) (Nakajima et al., 1992a), betaenone E (**8**) (Ichihara et al., 1983b), betaenone A (**9**) (Ichihara et al., 1983a), betaenone F (**10**) (Ichihara et al., 1983b), xylariolide D (**12**) (Tran et al., 2020), and xylariolide F (**13**) (Tran et al., 2020), respectively, based on the analysis of NMR spectra and comparison of literature.

*Neocamarosporium betae* is often found as a plant pathogen or endophyte on different plants, from which many bioactive secondary metabolites with different carbon skeletons have been isolated (Xu et al., 2021). The structure of compound **1** possesses a unique 5,6-dihydro-4*H*,7*H*-2,6-methanopyrano[4,3-*d*][1,3]dioxocin-7-one skeleton, which is the first report in *Nature*. Based on the structural features of **1**, the biosynthetic pathway of **1** might originate from two polyketide hybrids. Spiciferones (**3–5**), spiciferinone (**6**), and spicifernin (containing the core skeleton of 2) were co-isolated from the plant pathogen *Cochliobolus spicifer* Nelson, which causes leaf-spot disease in wheat (Nakajima et al., 1991; Nakajima et al., 1992a). Based on isotope-labeling experiments, Nakajima et al. proposed a biogenetic pathway for spicifernin (Nakajima et al., 1992b). Given that compounds **2–6** were isolated from *N. betae*, the biosynthetic pathways for compounds **2–6** might be the same as that of spicifernin (**Figure S35**). Betaenones (containing the core skeleton of compounds **7–10**) act as phytotoxins and were isolated from the pathogenic fungus *P. betae* Fr., which causes leaf-spot disease in sugar beet, inducing chlorosis in host plant leaves (Ichihara et al., 1983a; Ichihara et al., 1983b; Oikawa et al., 1984b). The structural features of betaenones (decalin scaffold) indicate that the core skeleton was formed by a Diels–Alder reaction ([4 + 2] cycloaddition). The biosynthesis of betaenone B was examined through isotope-labeling experiments, which revealed that the carbon skeleton originated from eight acetate units through a polyketide pathway, and the methyl groups were derived from methionine (Oikawa et al., 1984a). Recently, the gene cluster of betaenones was identified. The highly reducing polyketide synthase gene with a *trans*-acting enoyl reductase domain was heterologously expressed to form a decalin scaffold. In addition, a series of post-modification oxidative enzymes were investigated, which allowed for the reconstitution of the betaenone biosynthetic machinery (Ugai et al., 2015). Compound **11** is an analog of xylariolide D (**12**) and xylariolide F (**13**), which were recently isolated from the soil-derived fungus *Dictyosporium digitatum* (Tran et al., 2020). The possible biosynthetic pathways of **1–13** are shown in **Figure S35**.

The cytotoxic activities of compounds (1–13) were evaluated against three cancer cell lines (MCF-7, B16, and HepG2). Compounds **1**, **3**, **4**, and **9** displayed strong inhibitory activity with IC_50_ values of 1.12 ± 0.02, 1.02 ± 0.26, 2.35 ± 0.05, and 1.42 ± 0.15 μM, respectively, against HepG2 cells, compared with the positive control. Compounds **7–9** possessed moderate inhibitory activity against B16 cells (**Table 3**). Betaenones have been mainly isolated from the plant pathogenic fungi *Stemphylium botryosum* and *P. betae* (Barash et al., 1982; Barash et al., 1983; Ichihara et al., 1983a; Ichihara et al., 1983b; Manulis et al., 1984; Oikawa et al., 1984b). Stemphyloxin, the first betaenone analog from *S. botryosum*, produces the necrotic and chlorotic blighting symptoms on tomato leaves (Barash et al., 1982; Barash et al., 1983; Manulis et al., 1984). Betaenones A–F, isolated from *P. betae*, exhibited phytotoxicity, resulting in the wilting of the host sugar beet plant (Ichihara et al., 1983a; Ichihara et al., 1983b; Oikawa et al., 1984b). The phytotoxic effects of compounds **2–5** and **7–13** were evaluated on green foxtail and corn leaves. Most compounds (**2**, **4–5**, **7–9**, and **11–13**) displayed phytotoxicity against foxtail leaves by producing lesions; however, compounds **2–5** and **7–13** did not show phytotoxicity against corn leaves (**Figure S36**). Although the mechanism of action of these phytotoxic metabolites (**2**, **4–5**, **7–9**, and **11–13**) must be elucidated in the future, they can be used as potent herbicides in corn cultivation.

**Table 3:** Cytotoxicity (IC_50_, μM) of compounds **1−13** against cancer cell lines.

Compound	MCF-7	B16	HepG2
**1**	≥100	≥100	1.12 ± 0.02
**2**	26.60 ± 3.32	≥100	4.88 ± 0.19
**3**	≥100	≥100	1.02 ± 0.26
**4**	≥100	≥100	2.35 ± 0.05
**5**	≥100	≥100	≥100
**6**	≥100	≥100	≥100
**7**	35.16 ± 1.28	4.42 ± 0.62	7.06 ± 0.15
**8**	≥100	4.73 ± 1.17	≥100
**9**	≥100	24.05 ± 0.69	1.42 ± 0.15
**10**	≥100	≥100	5.66 ± 0.43
**11**	≥100	≥100	≥100
**12**	≥100	≥100	≥100
**13**	≥100	≥100	≥100
*cis*-platin	6.57 ± 0.22	17.95 ± 3.16	3.69 ± 0.74

## 4 Conclusion

A chemical investigation was conducted on the secondary metabolites produced by the endophytic fungus *N. betae* that was isolated from two desert plants. A total of 13 polyketides (**1–13**), including four undescribed molecules (**1**, **2**, **7**, and **11**), were purified. The new metabolites were examined using HR-ESI-MS, UV spectroscopy, IR spectroscopy, NMR, and CD. The different carbon skeletons of compounds **1–13** enriched the structural diversity of secondary metabolites from *N. betae*. The possible biosynthetic pathways were suggested according to the structural features of compounds **1–13**. In addition, compounds **1**, **3**, **4**, and **9** exhibited strong cytotoxicity toward HepG2 cancer cells, and compounds **2**, **4–5**, **7–9**, and **11–13** displayed phytotoxic activities against foxtail leaves. The results in this report provide further evidence that endophytic fungi inhabiting specific biotopes, such as desert plants, could be new sources of novel secondary metabolites with different biological activities.

## Data availability statement

The datasets presented in this study can be found in online repositories. The names of the repository/repositories and accession number(s) can be found in the article/**Supplementary Material**.

## Author contributions

SY and GD conceived and designed the study. PL and YT performed the experiments and collected the experimental data. PL, YT, Y-DW, and QL evaluated the activities of all the isolates. JY and D-AS provided funding and analyzed the original draft. X-KX and B-DS provided resources. PL, YT, S-XY, and GD wrote the first draft of the manuscript. PL, YT, and GD revised the manuscript. All authors contributed to the article and approved the submitted version.
