# Supplementary material for: Bioactive secondary metabolites from endophytic strains of Neocamarosporium betae collected from desert plants
Source: Front Plant Sci. 2023 Mar 17;14:1142212. doi: 10.3389/fpls.2023.1142212 (PMC10063976; doi:10.3389/fpls.2023.1142212)

Supplementary Material

Bioactive Secondary Metabolites from Endophytic Strains of Neocamarosporium betae Collected from Desert Plants

Peng Liu[1,2†], Yue Tan[1†], Jian Yang[3], Yan-Duo Wang[1], Qi Li[1], Bing-Da Sun[4], Xiao-Ke Xing[1], Di-An Sun[1], Sheng-Xiang Yang[2*] and Gang Ding[1*]

*** Correspondence:** Sheng-Xiang Yang: shengxiangyang2000@163.com; Gang Ding: gding@implad.ac.cn

Supplementary Figures

CONTENTS

Figure S1. ^{1}H NMR spectrum (500 MHz) of **1** in $CDCl_3$. 3

Figure S2. ^{13}C NMR spectrum (150 MHz) of **1** in $CDCl_3$. 3

Figure S3. HSQC spectrum (500 MHz) of **1** in $CDCl_3$. 4

Figure S4. ^{1}H-^{1}H COSY spectrum (500 MHz) of **1** in $CDCl_3$. 4

Figure S5. HMBC spectrum (500 MHz) of **1** in $CDCl_3$. 5

Figure S6. ROESY spectrum (500 MHz) of **1** in $CDCl_3$. 5

Figure S7. IR spectrum of **1**. 6

Figure S8. UV spectrum of **1** in MeOH. 6

Figure S9. HRESIMS spectrum of **1**. 7

Figure S10. ^{1}H NMR spectrum (500 MHz) of **2** in $CDCl_3$. 7

Figure S11. ^{13}C NMR spectrum (150 MHz) of **2** in $CDCl_3$. 8

Figure S12. HSQC spectrum (500 MHz) of **2** in $CDCl_3$. 8

Figure S13. ^{1}H-^{1}H COSY spectrum (500 MHz) of **2** in $CDCl_3$. 9

Figure S14. HMBC spectrum (500 MHz) of **2** in $CDCl_3$. 9

Figure S15. IR spectrum of **2**. 10

Figure S16. UV spectrum of **2** in MeOH. 10

Figure S17. HRESIMS spectrum of **2**. 10

Figure S18. ^{1}H NMR spectrum (600 MHz) of **7** in $CDCl_3$. 11

Figure S19. ^{13}C NMR spectrum (150 MHz) of **7** in $CDCl_3$. 12

Figure S20. HSQC spectrum (600 MHz) of **7** in $CDCl_3$. 12

Figure S21. ^{1}H-^{1}H COSY spectrum (600 MHz) of **7** in $CDCl_3$. 13

Figure S22. HMBC spectrum (600 MHz) of **7** in $CDCl_3$. 13

Figure S23. CD spectrum of **7** and **10**. 14

Figure S24. IR spectrum of **7**. 14

Figure S25. UV spectrum of **7** in MeOH. 15

Figure S26. HRESIMS spectrum of **7**. 15

Figure S27. ^{1}H NMR spectrum (600 MHz) of **11** in $CDCl_3$. 16

Figure S28. ^{13}C NMR spectrum (150 MHz) of **11** in $CDCl_3$. 16

Figure S29. HSQC spectrum (600 MHz) of **11** in $CDCl_3$. 17

Figure S30. ^{1}H-^{1}H COSY spectrum (600 MHz) of **11** in $CDCl_3$. 17

Figure S31. HMBC spectrum (600 MHz) of **11** in $CDCl_3$. 18

Figure S32. IR spectrum of **11**. 18

Figure S33. UV spectrum of **11** in MeOH. 19

Figure S34. HRESIMS spectrum of **11**. 19

Figure S35. The possible biosynthetic pathways of 1–13. 20

Figure S36. Phytotoxic effects on foxtail and corn leaves of **2–5**, and **7–13**. 21

Figure S1. ^{1}H NMR spectrum (500 MHz) of **1** in $CDCl_3$.

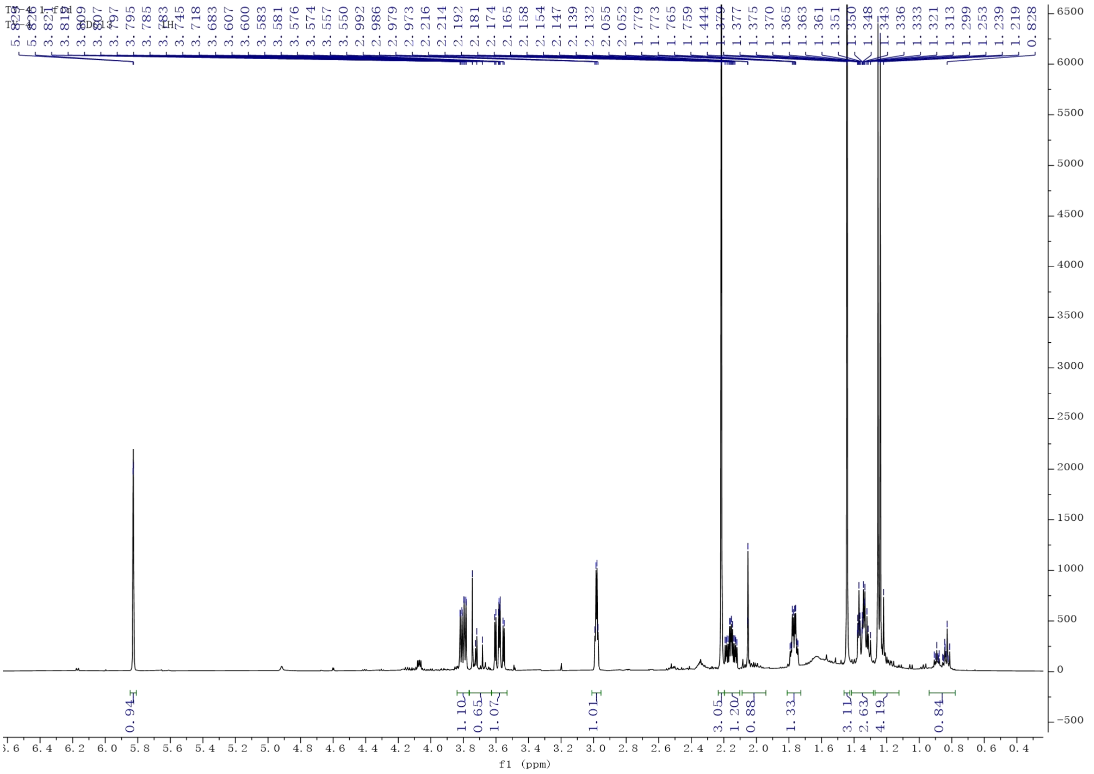

Figure S2. ^{13}C NMR spectrum (150 MHz) of **1** in $CDCl_3$.

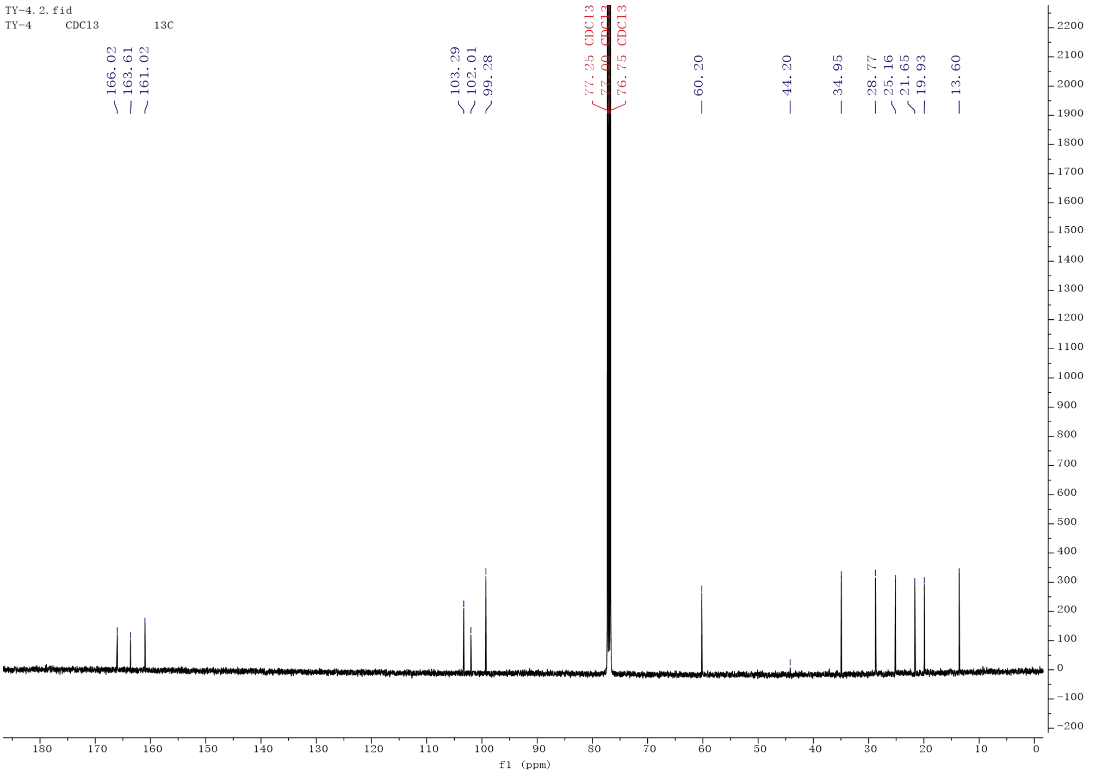

Figure S3. HSQC spectrum (500 MHz) of **1** in $CDCl_3$.

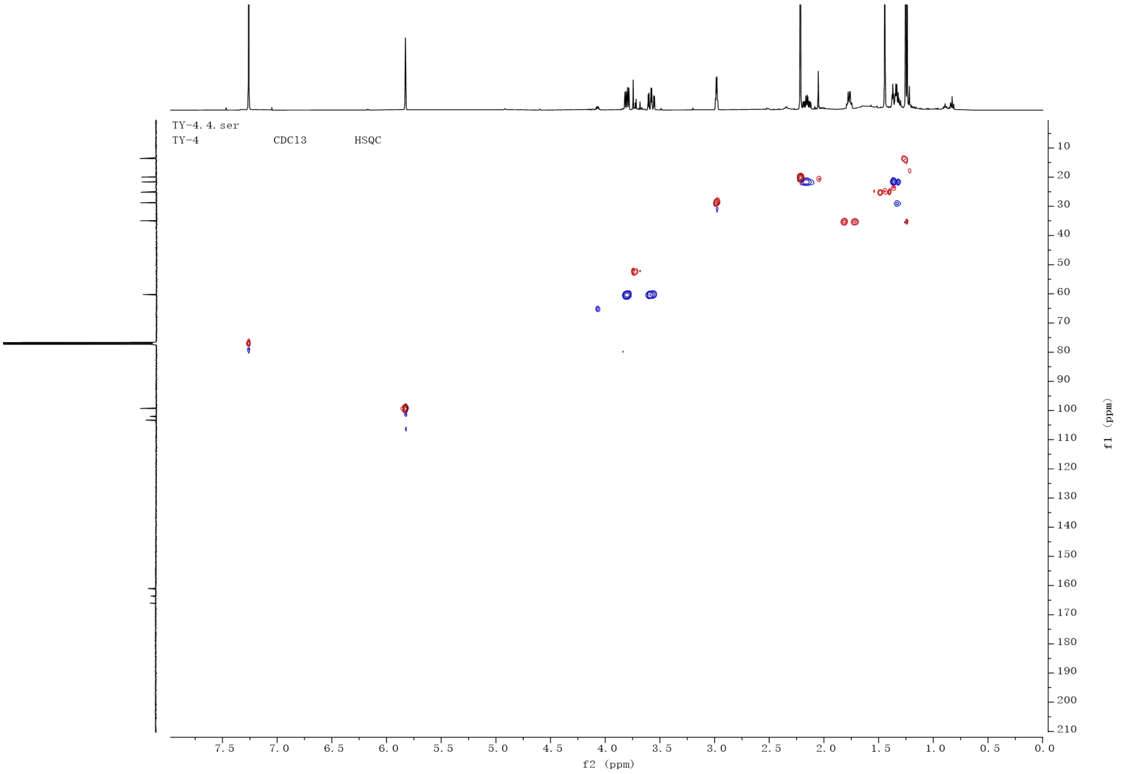

Figure S4. ^{1}H-^{1}H COSY spectrum (500 MHz) of **1** in $CDCl_3$.

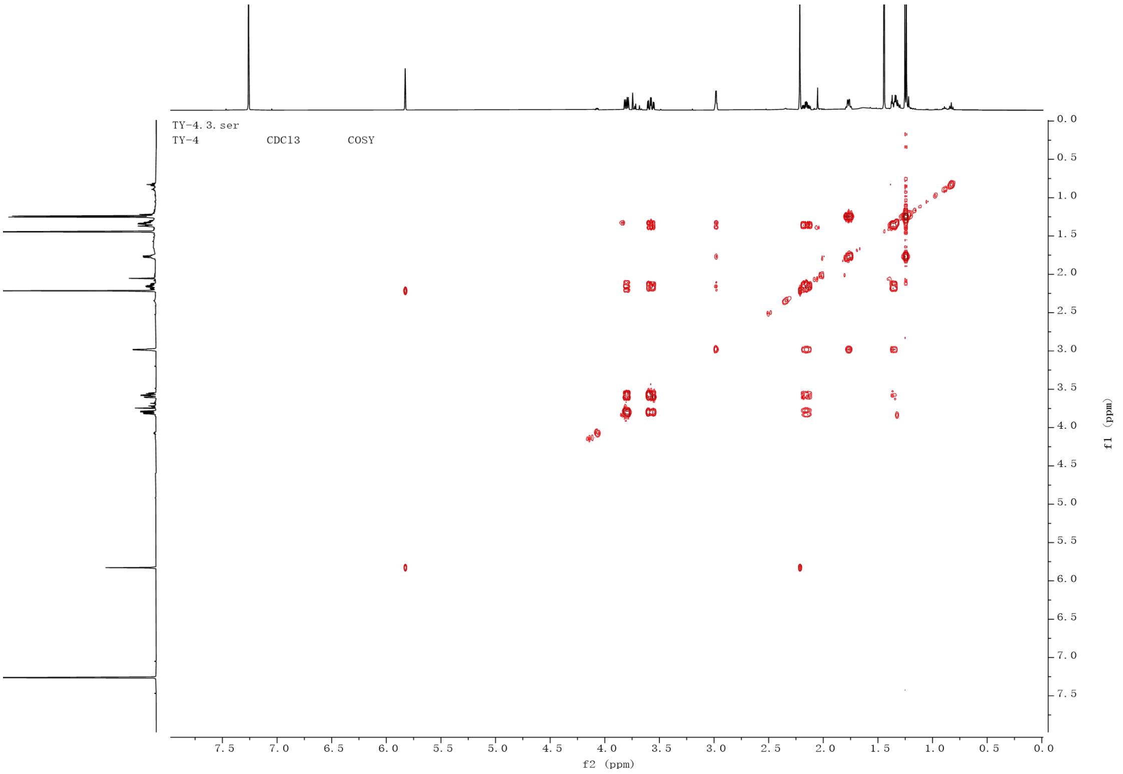

Figure S5. HMBC spectrum (500 MHz) of **1** in $CDCl_3$.

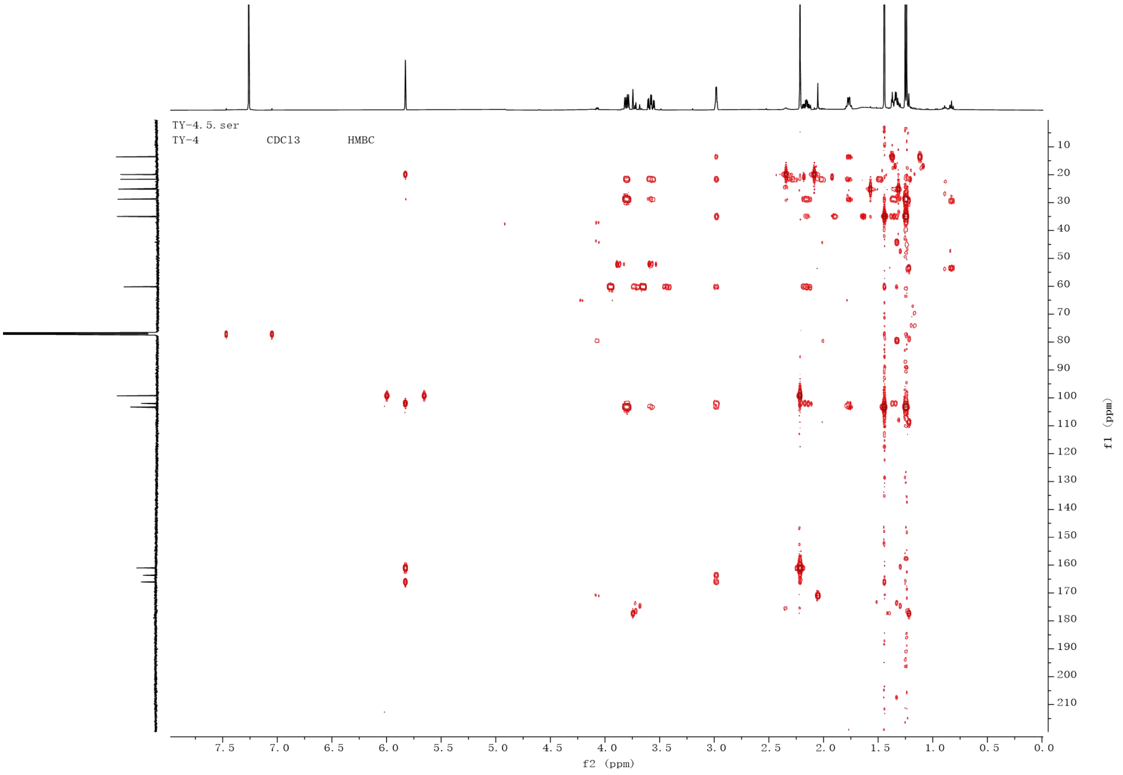

Figure S6. ROESY spectrum (500 MHz) of **1** in $CDCl_3$.

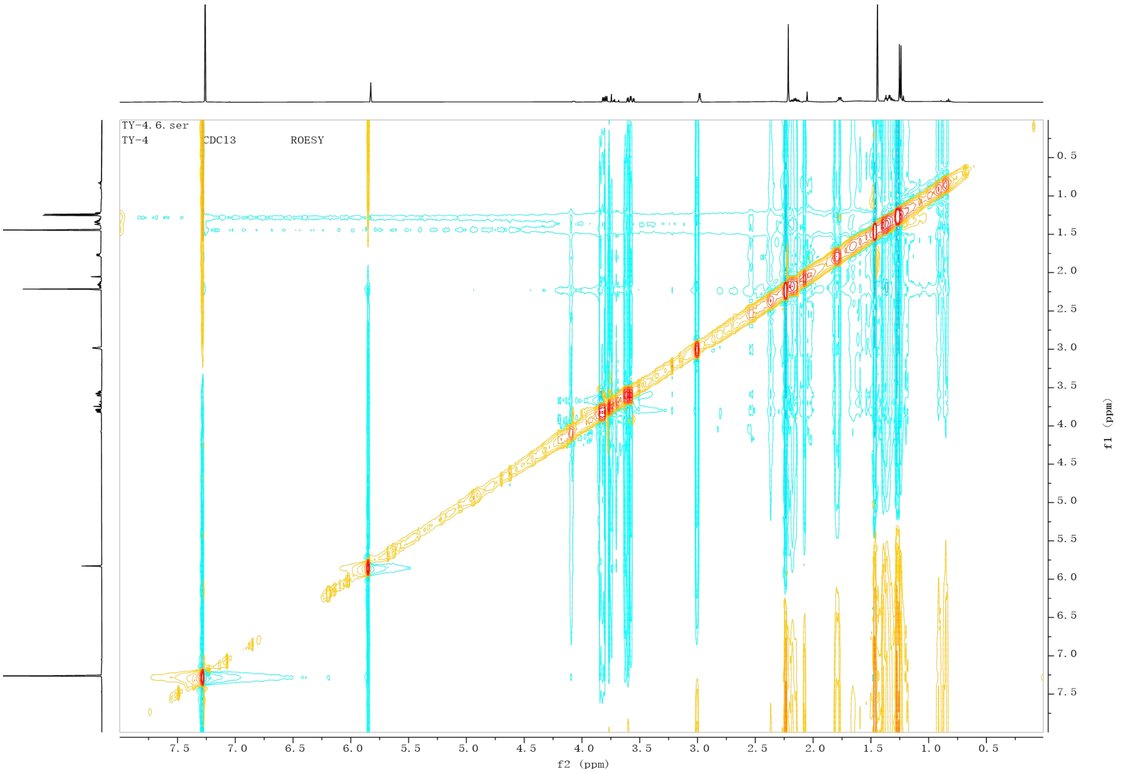

Figure S7. IR spectrum of **1**.

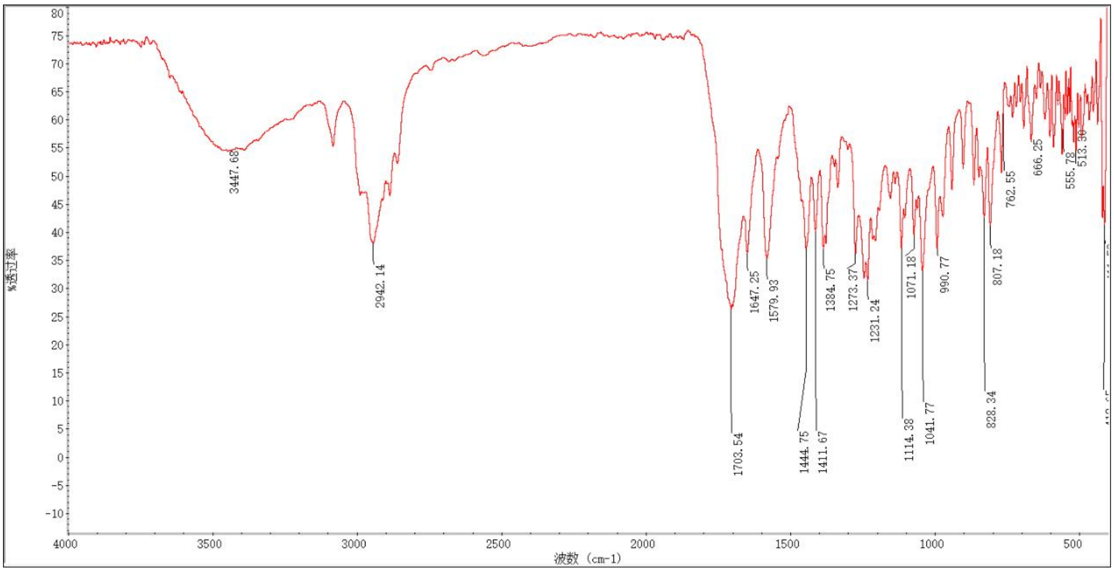

Figure S8. UV spectrum of **1** in MeOH.

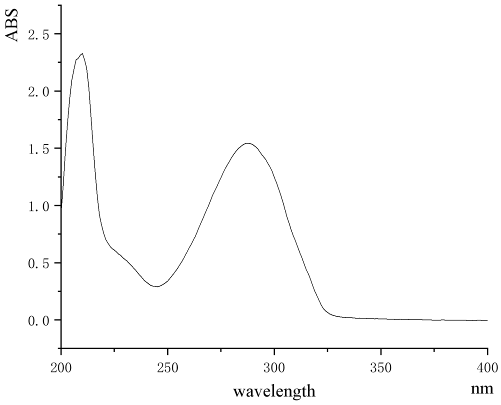

Figure S9. HRESIMS spectrum of **1**.

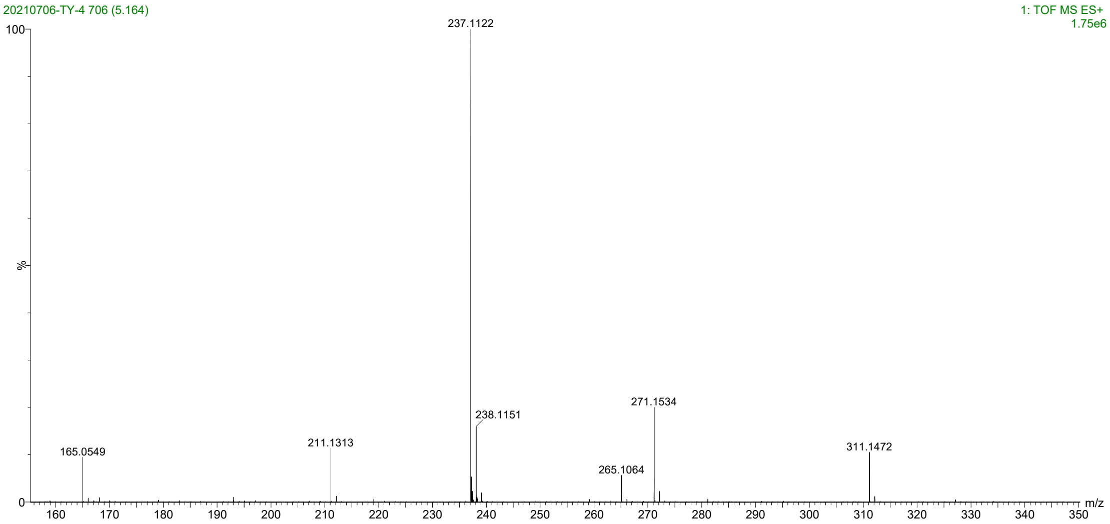

Figure S10. ^{1}H NMR spectrum (500 MHz) of **2** in $CDCl_3$.

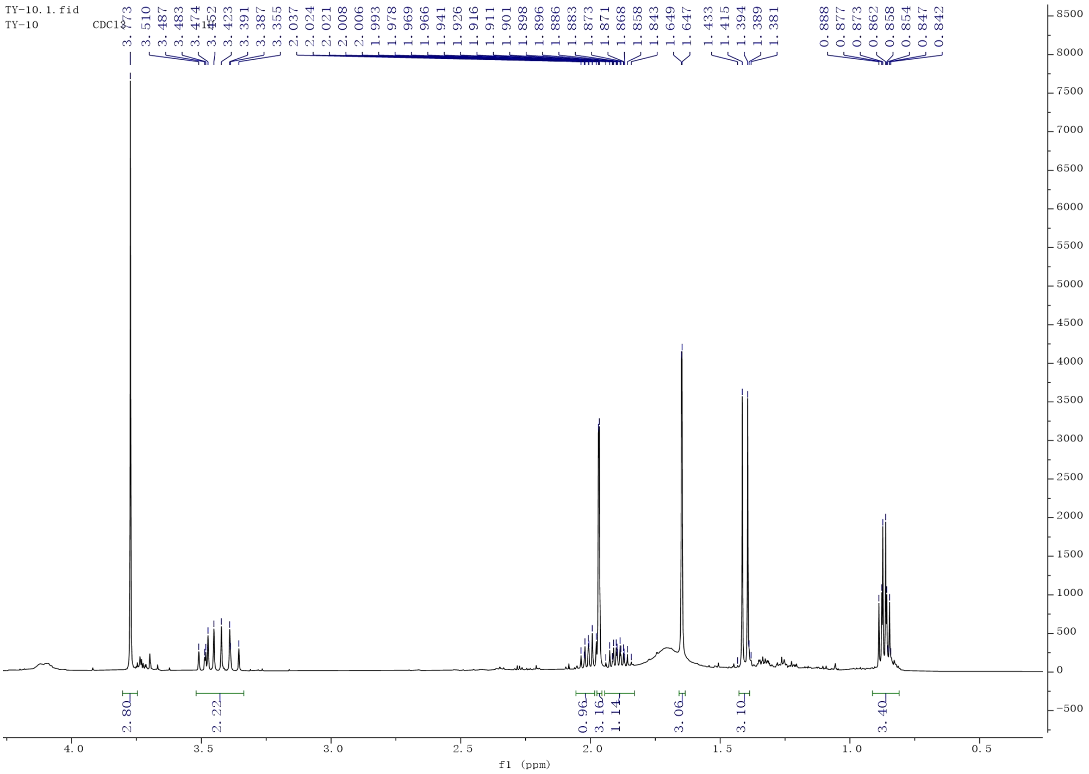

Figure S11. ^{13}C NMR spectrum (150 MHz) of **2** in $CDCl_3$.

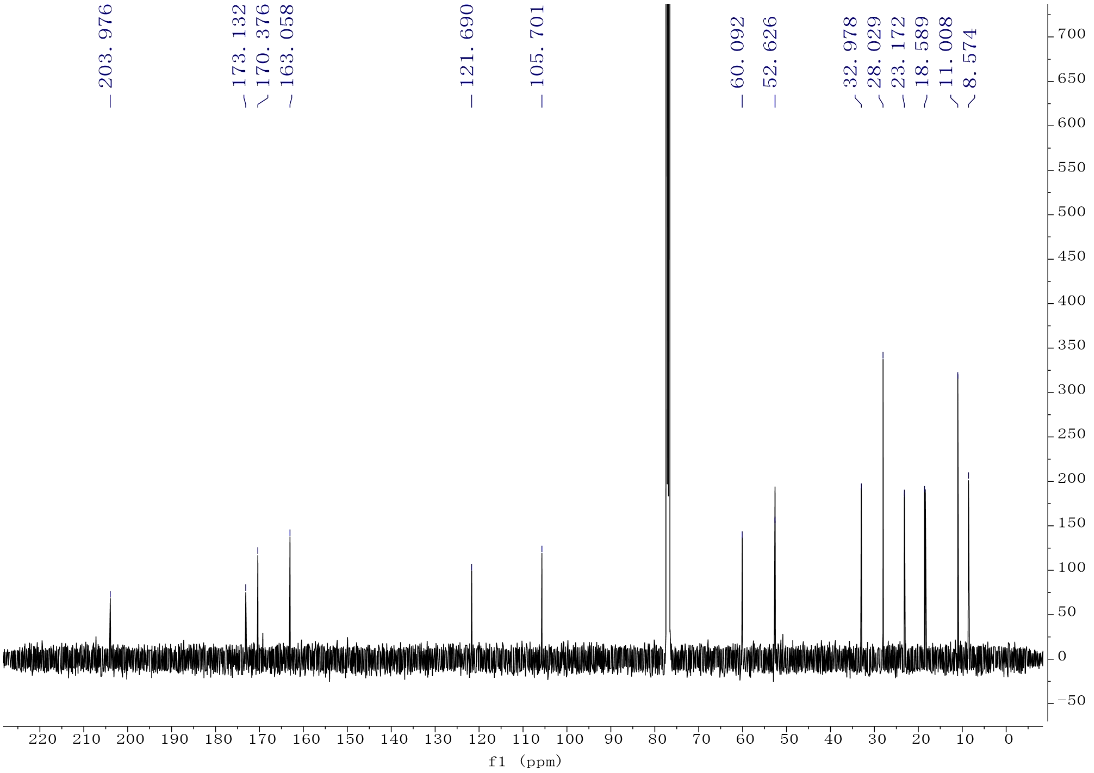

Figure S12. HSQC spectrum (500 MHz) of **2** in $CDCl_3$.

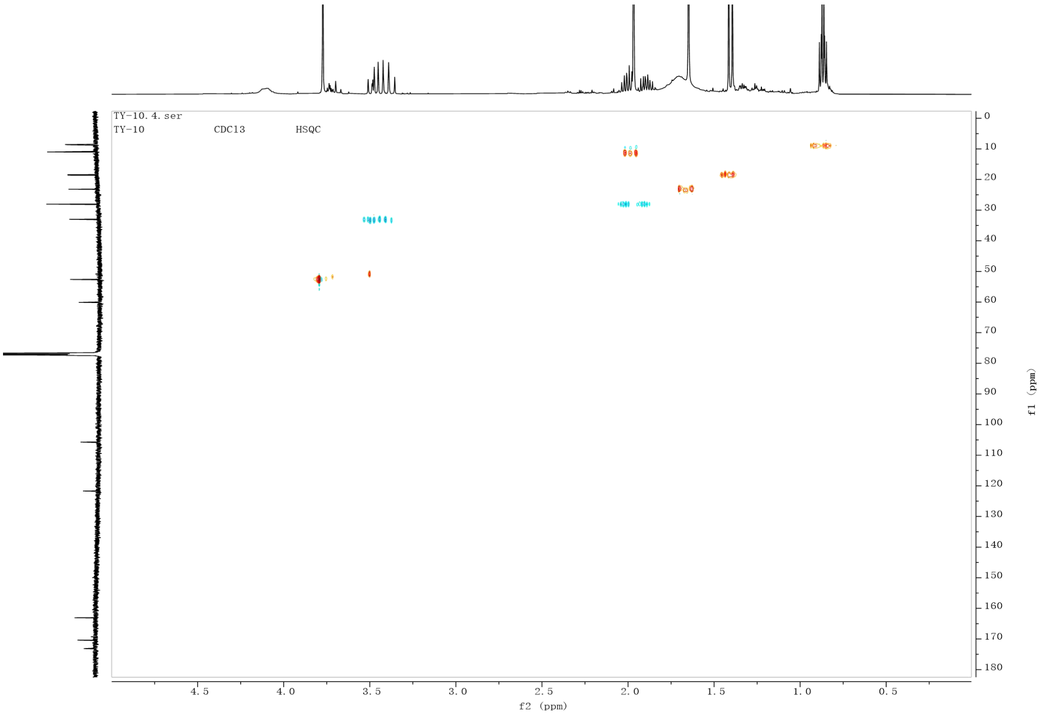

Figure S13. ^{1}H-^{1}H COSY spectrum (500 MHz) of **2** in $CDCl_3$.

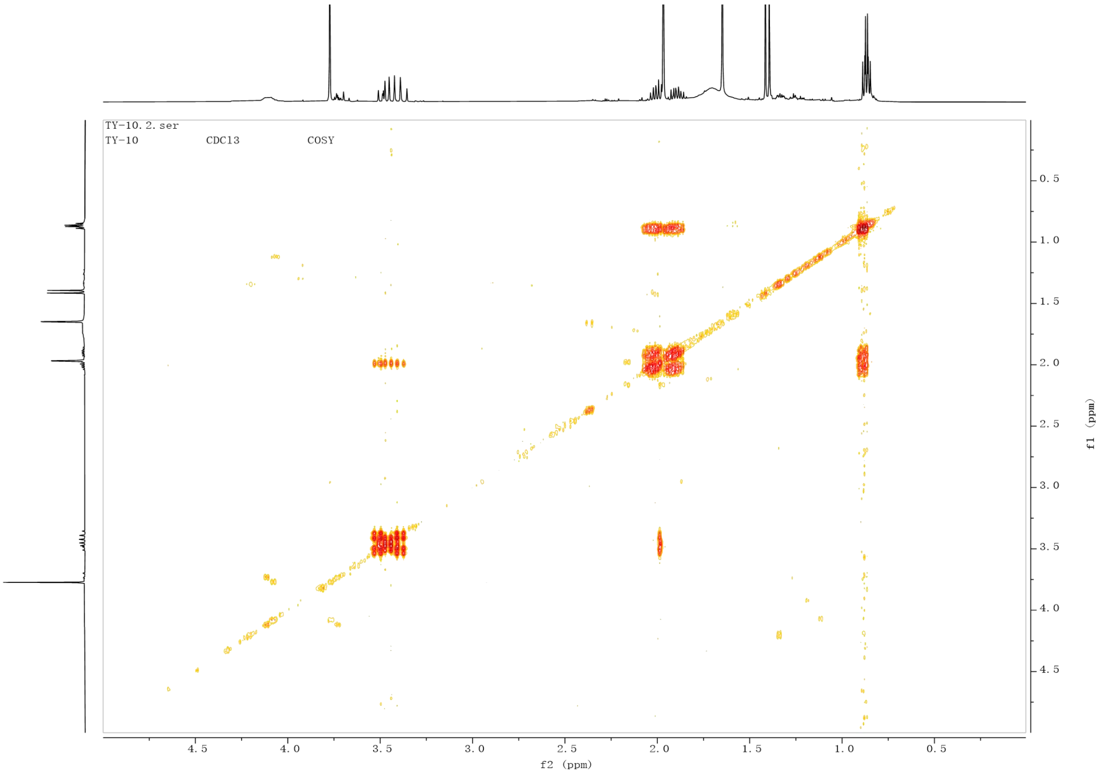

Figure S14. HMBC spectrum (500 MHz) of **2** in $CDCl_3$.

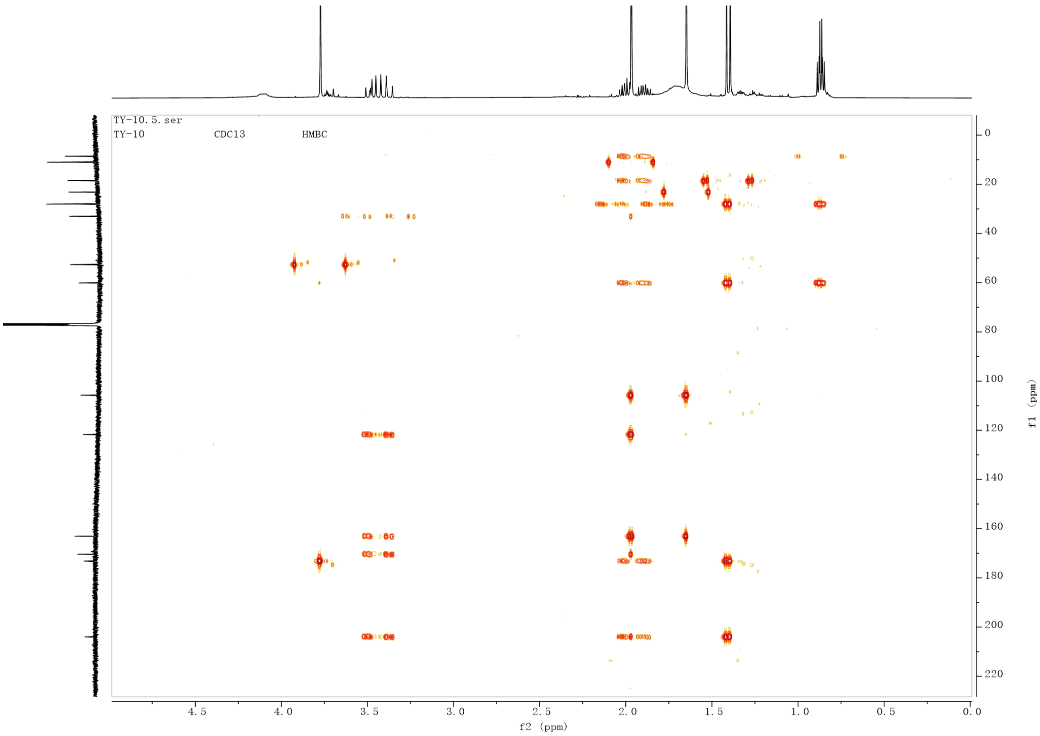

Figure S15. IR spectrum of **2**.

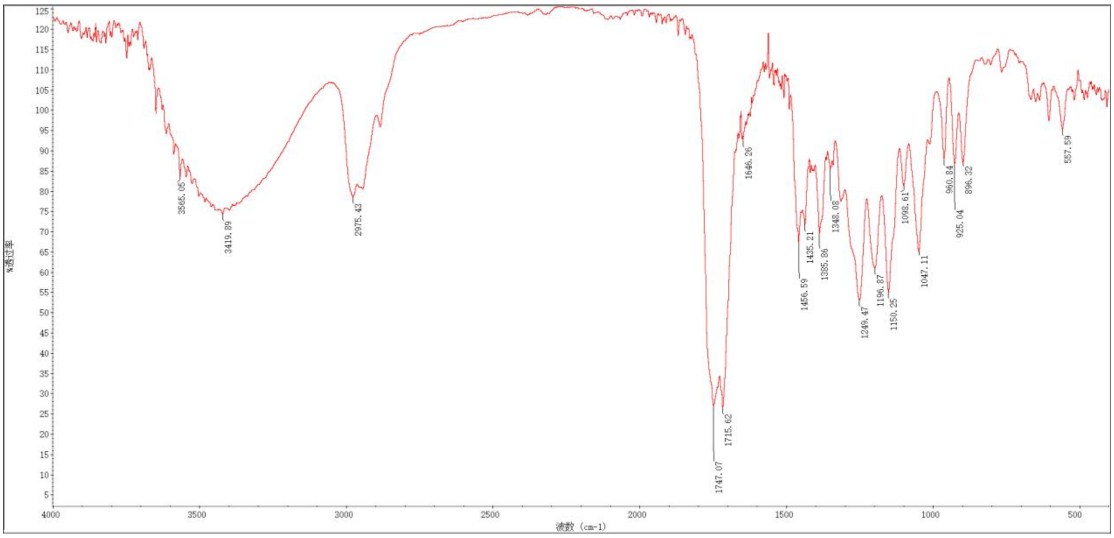

Figure S16. UV spectrum of **2** in MeOH.

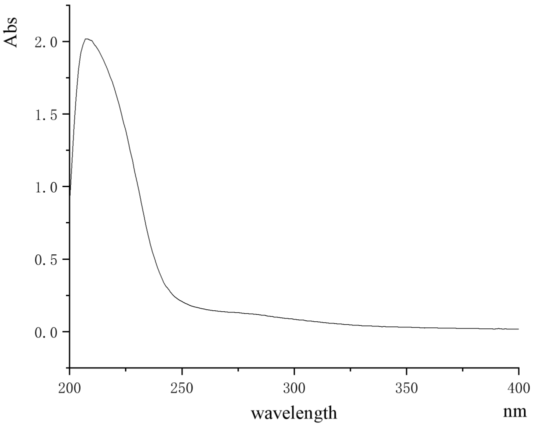

Figure S17. HRESIMS spectrum of **2**.

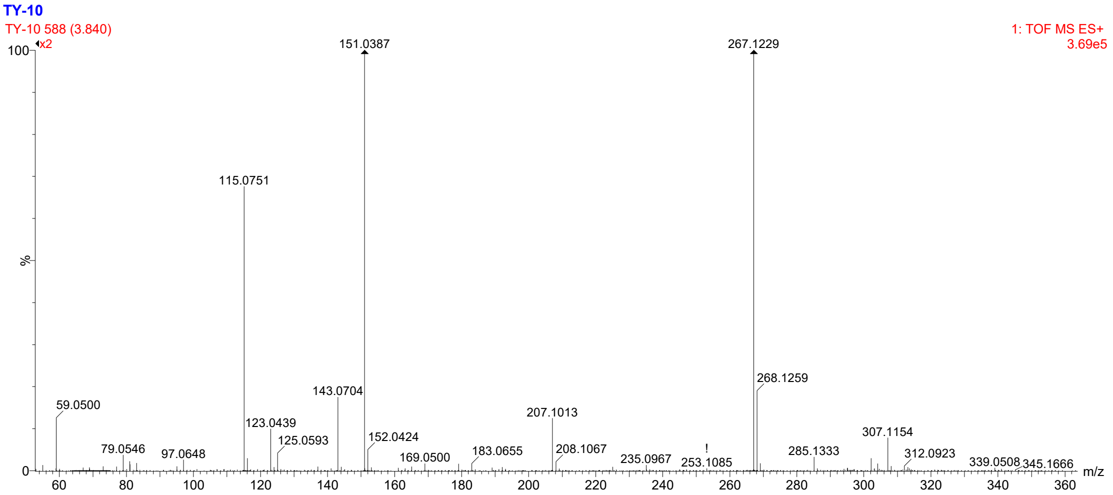

Figure S18. ^{1}H NMR spectrum (600 MHz) of **7** in $CDCl_3$.

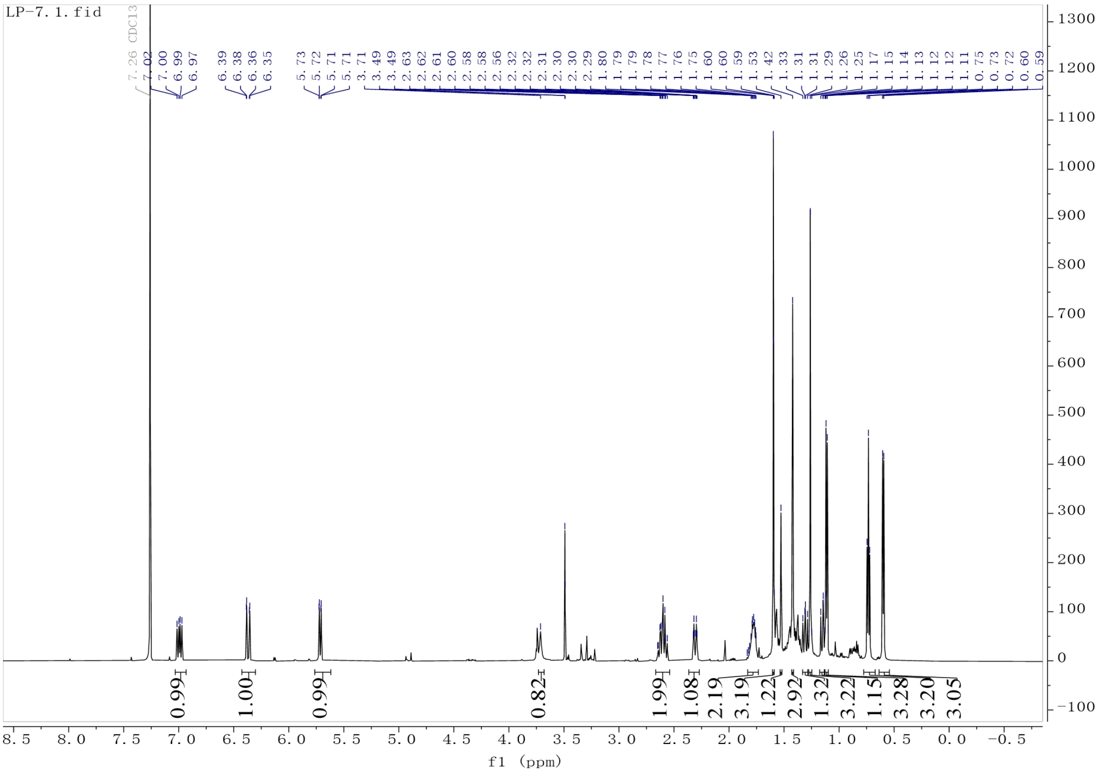

Figure S19. ^{13}C NMR spectrum (150 MHz) of **7** in $CDCl_3$.

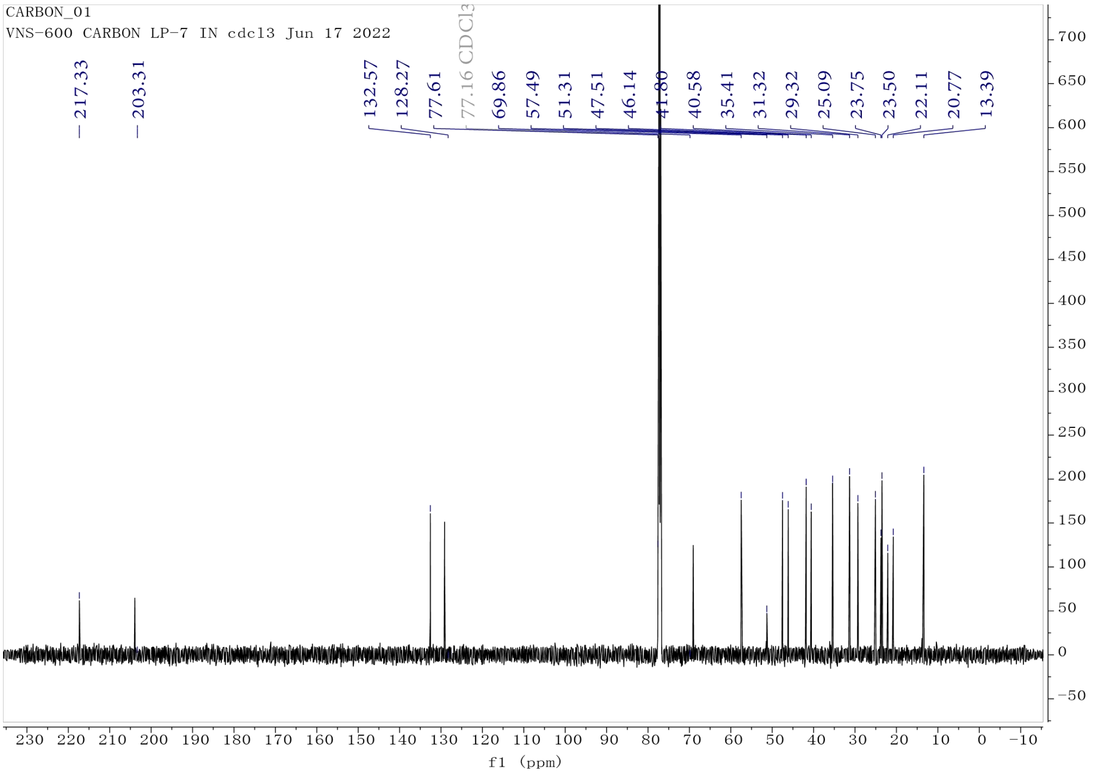

Figure S20. HSQC spectrum (600 MHz) of **7** in $CDCl_3$.

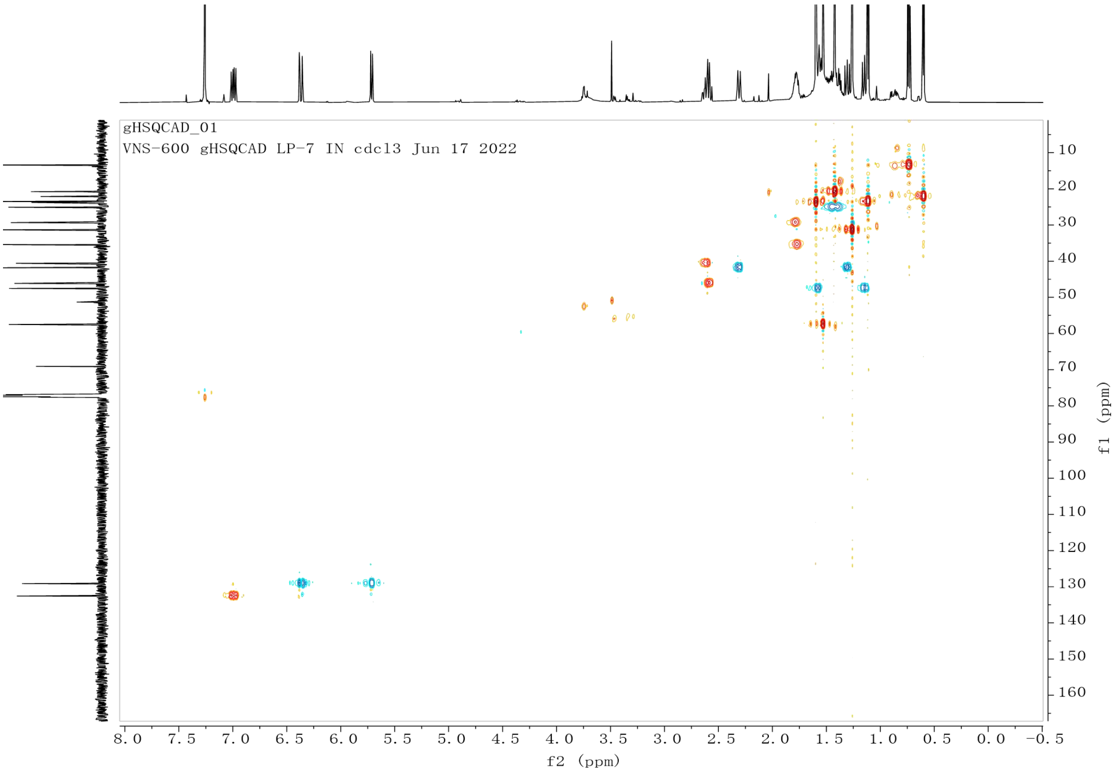

Figure S21. ^{1}H-^{1}H COSY spectrum (600 MHz) of **7** in $CDCl_3$.

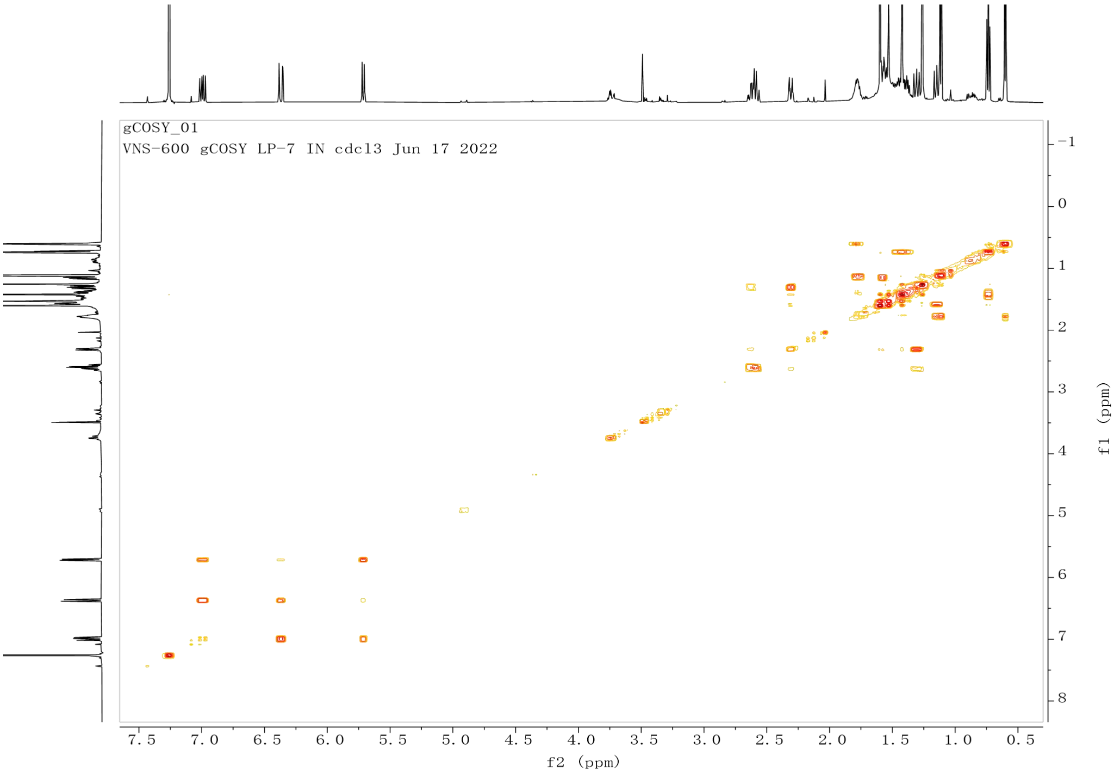

Figure S22. HMBC spectrum (600 MHz) of **7** in $CDCl_3$.

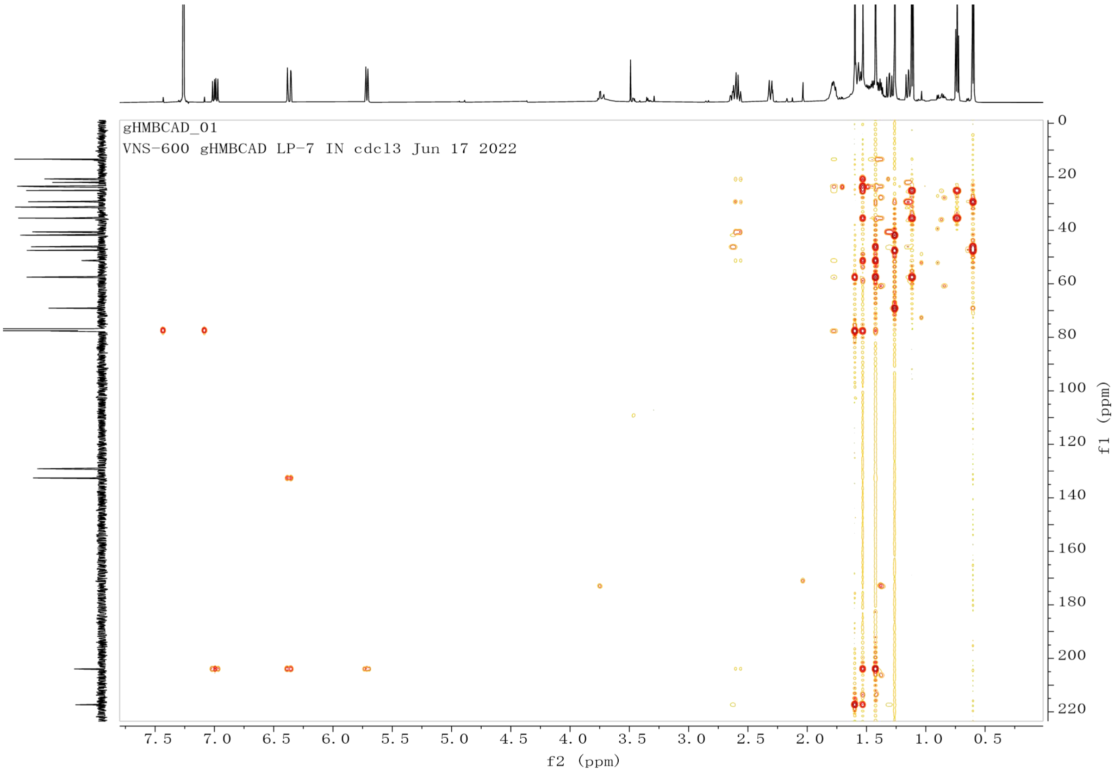

Figure S23. CD spectrum of **7** and **10**.

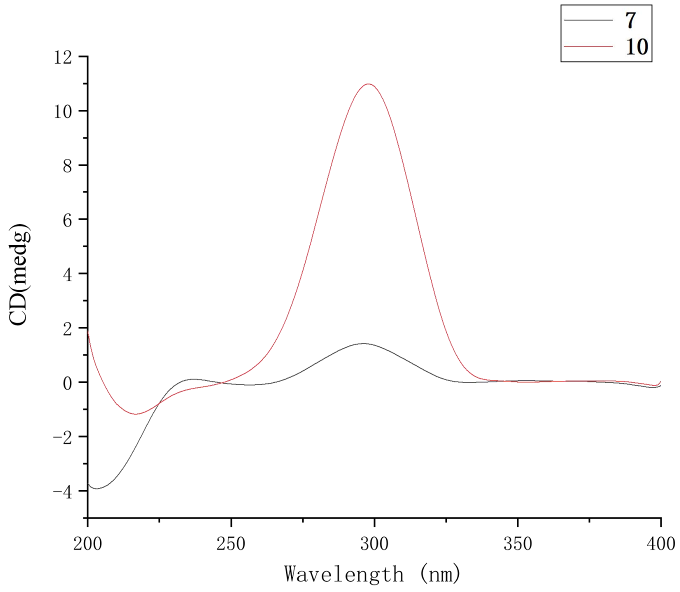

Figure S24. IR spectrum of **7**.

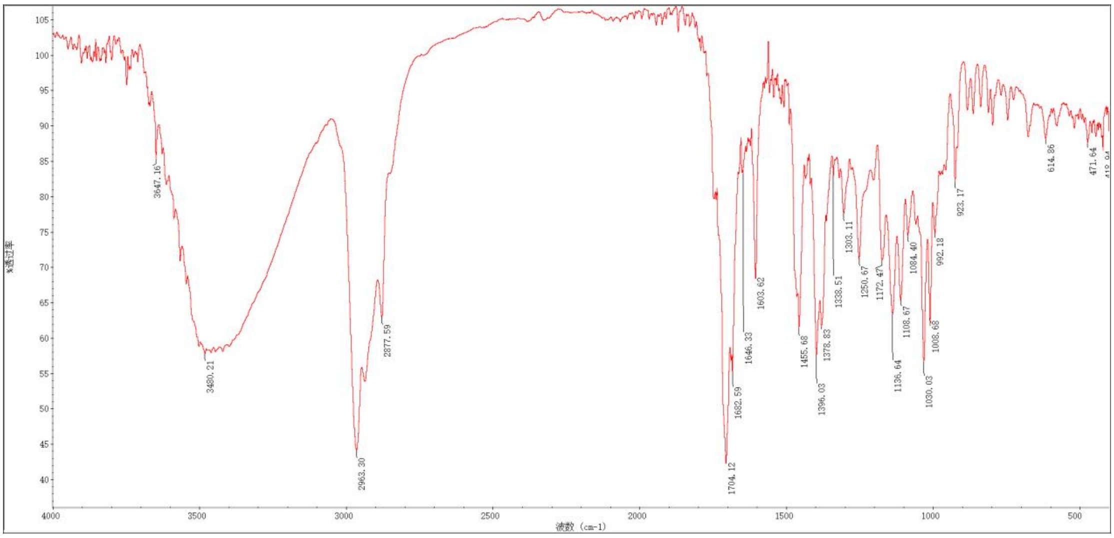

Figure S25. UV spectrum of **7** in MeOH.

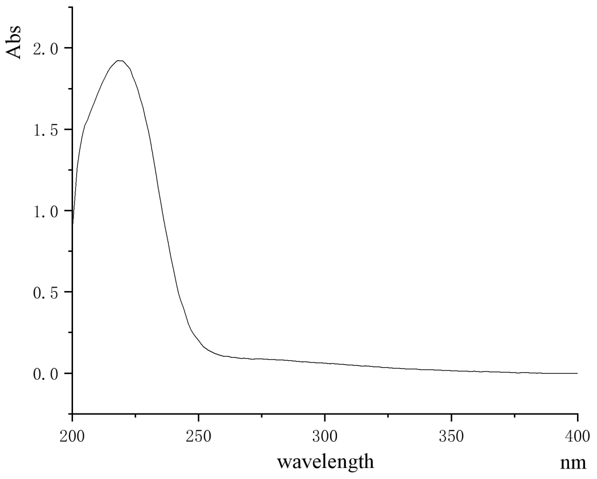

Figure S26. HRESIMS spectrum of **7**.

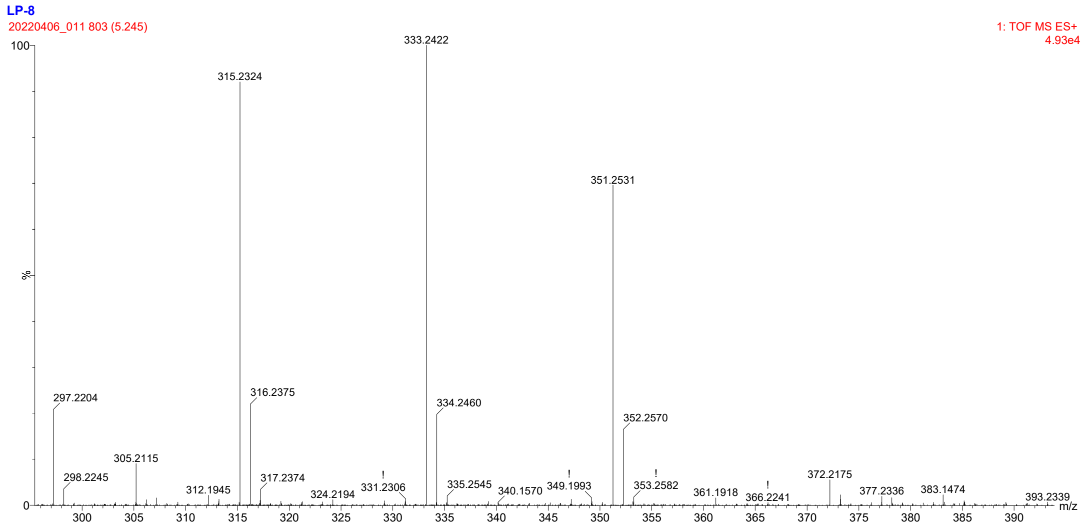

Figure S27. ^{1}H NMR spectrum (600 MHz) of **11** in $CDCl_3$.

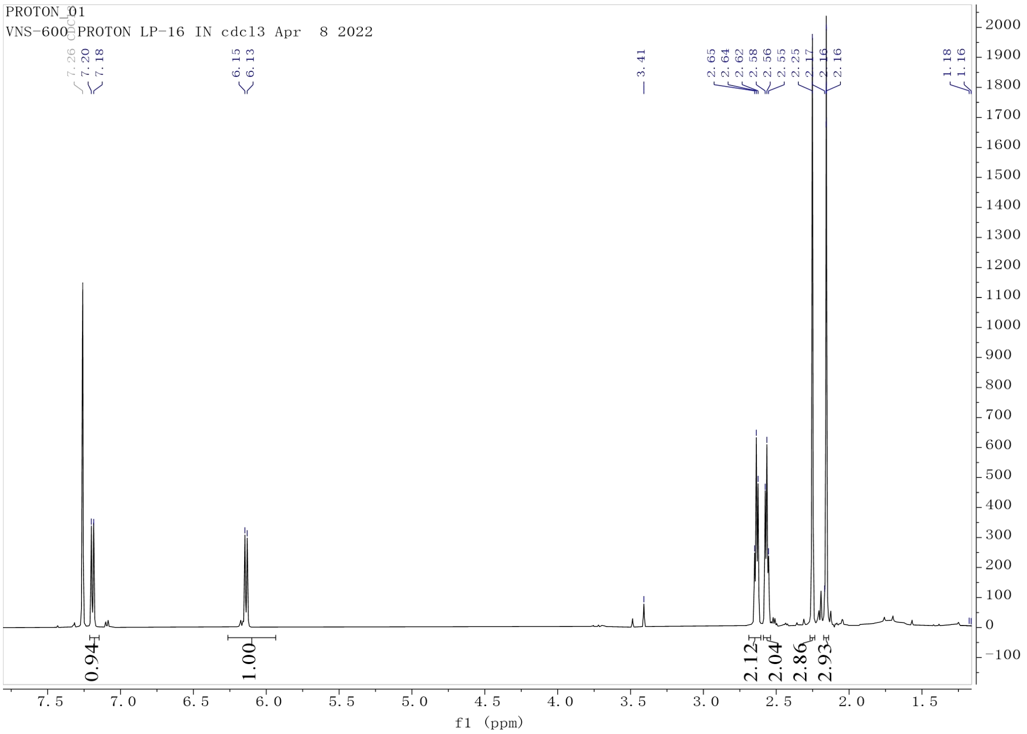

Figure S28. ^{13}C NMR spectrum (150 MHz) of **11** in $CDCl_3$.

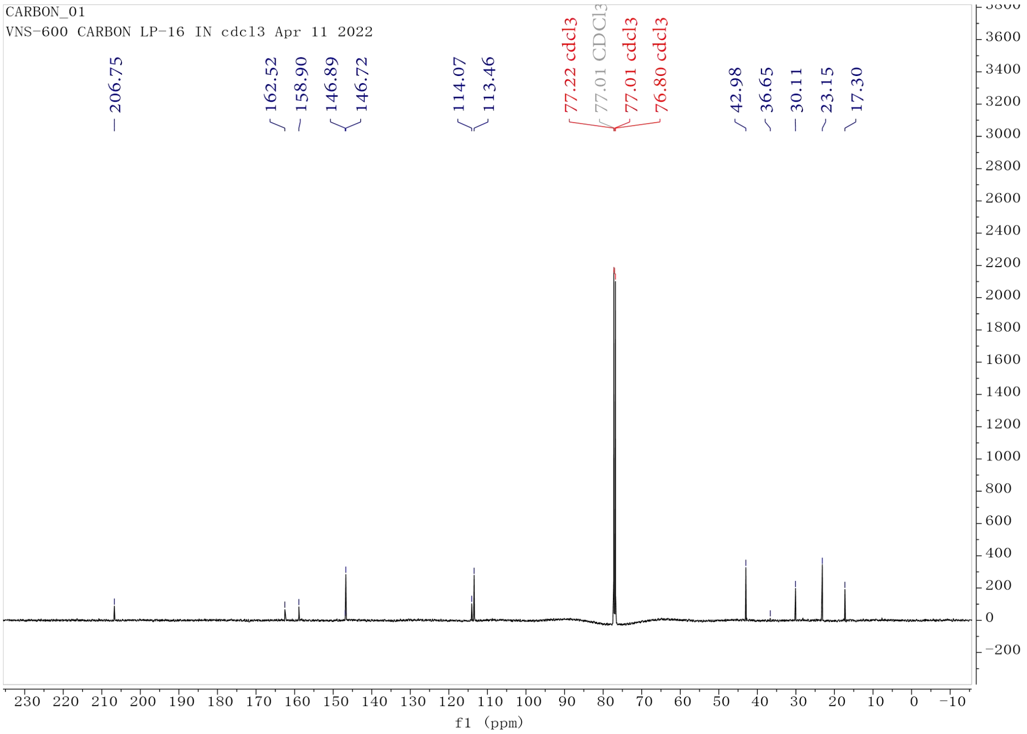

Figure S29. HSQC spectrum (600 MHz) of **11** in $CDCl_3$.

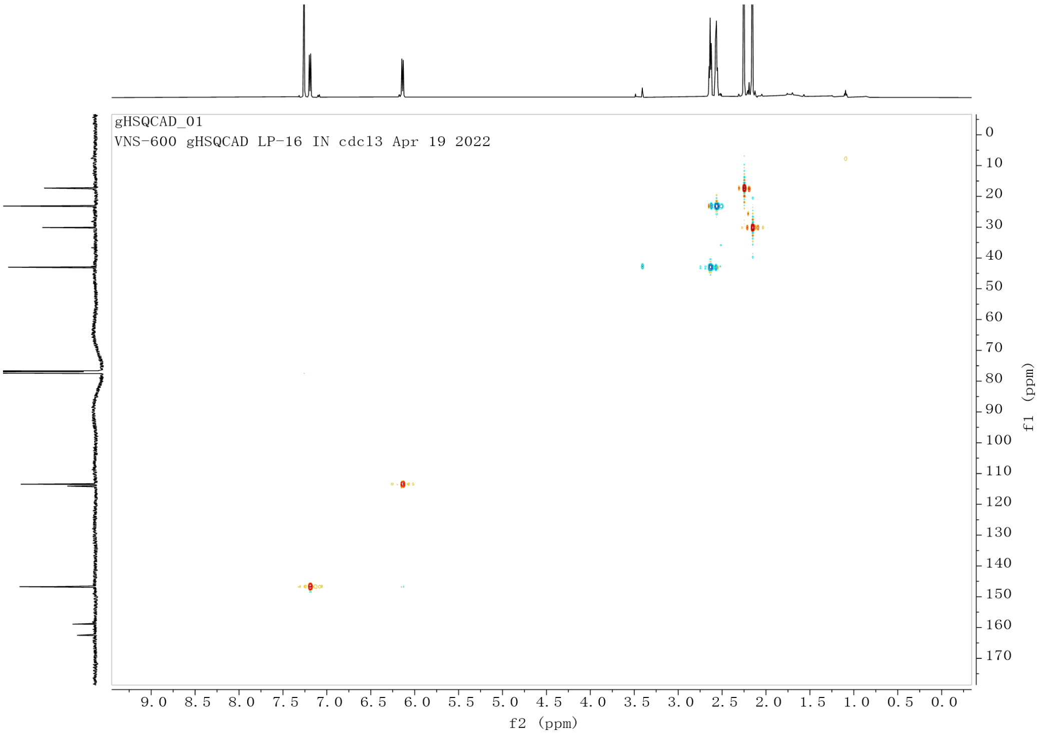

Figure S30. ^{1}H-^{1}H COSY spectrum (600 MHz) of **11** in $CDCl_3$.

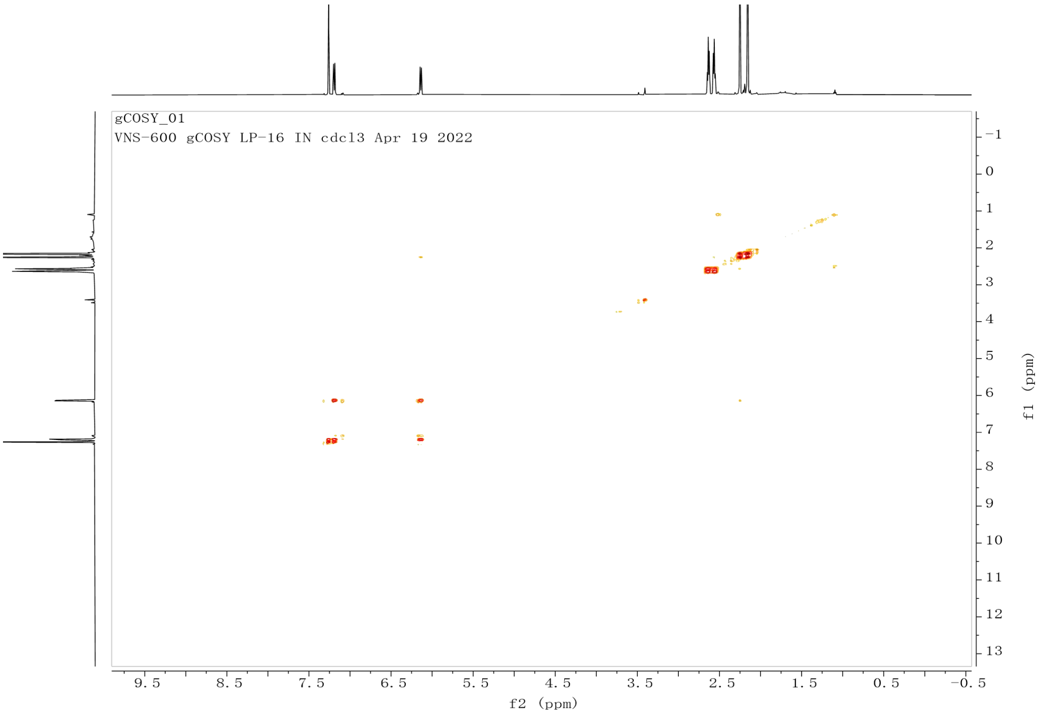

Figure S31. HMBC spectrum (600 MHz) of **11** in $CDCl_3$.

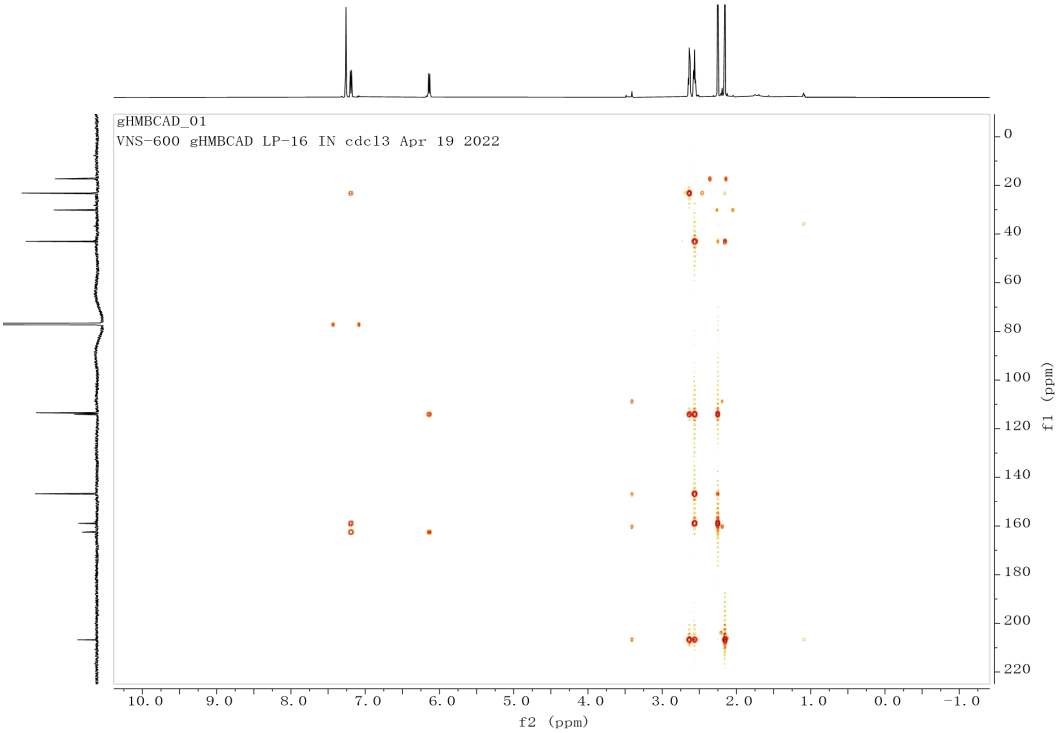

Figure S32. IR spectrum of **11**.

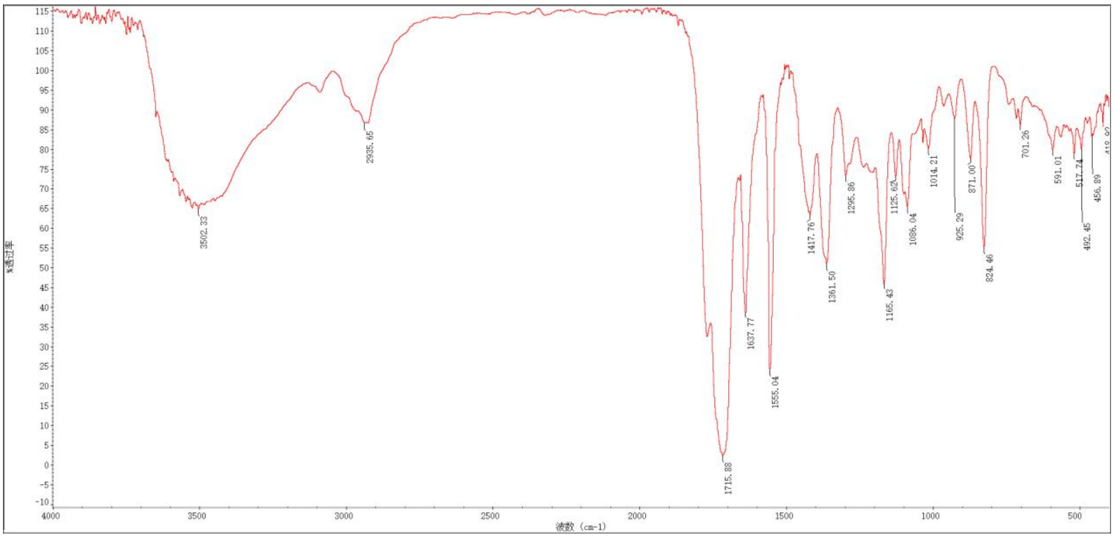

Figure S33. UV spectrum of **11** in MeOH.

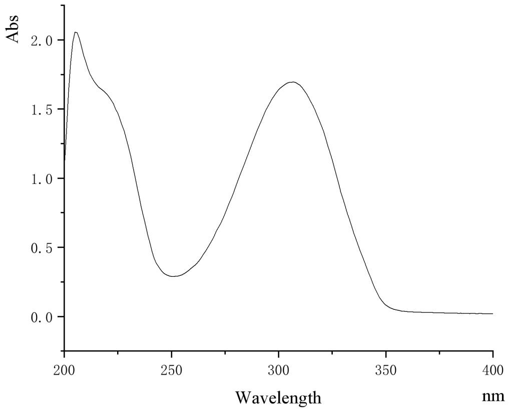

Figure S34. HRESIMS spectrum of **11**.

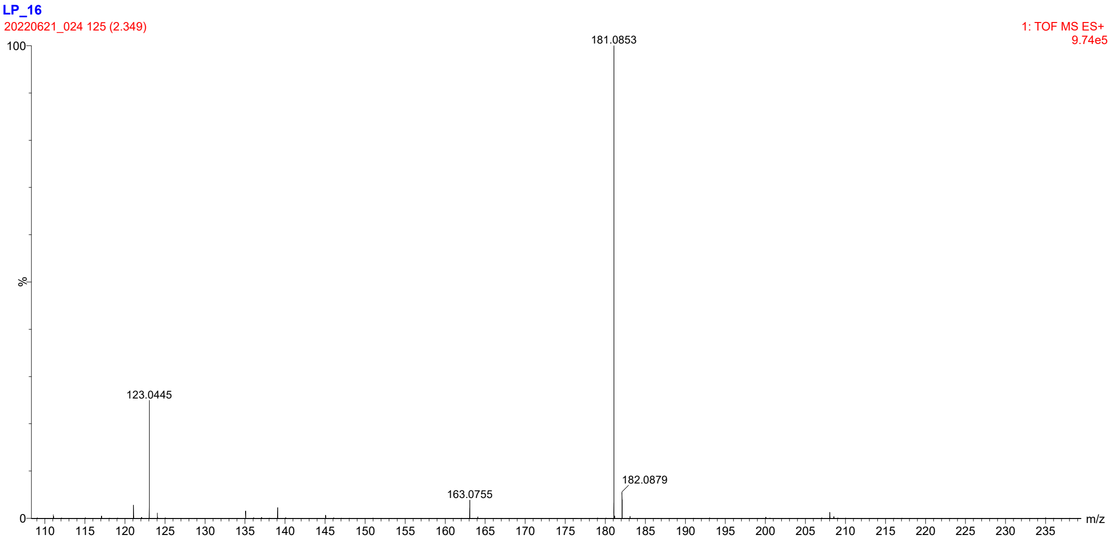

frontiers

Figure S35. The possible biosynthetic pathways of **1–13**.

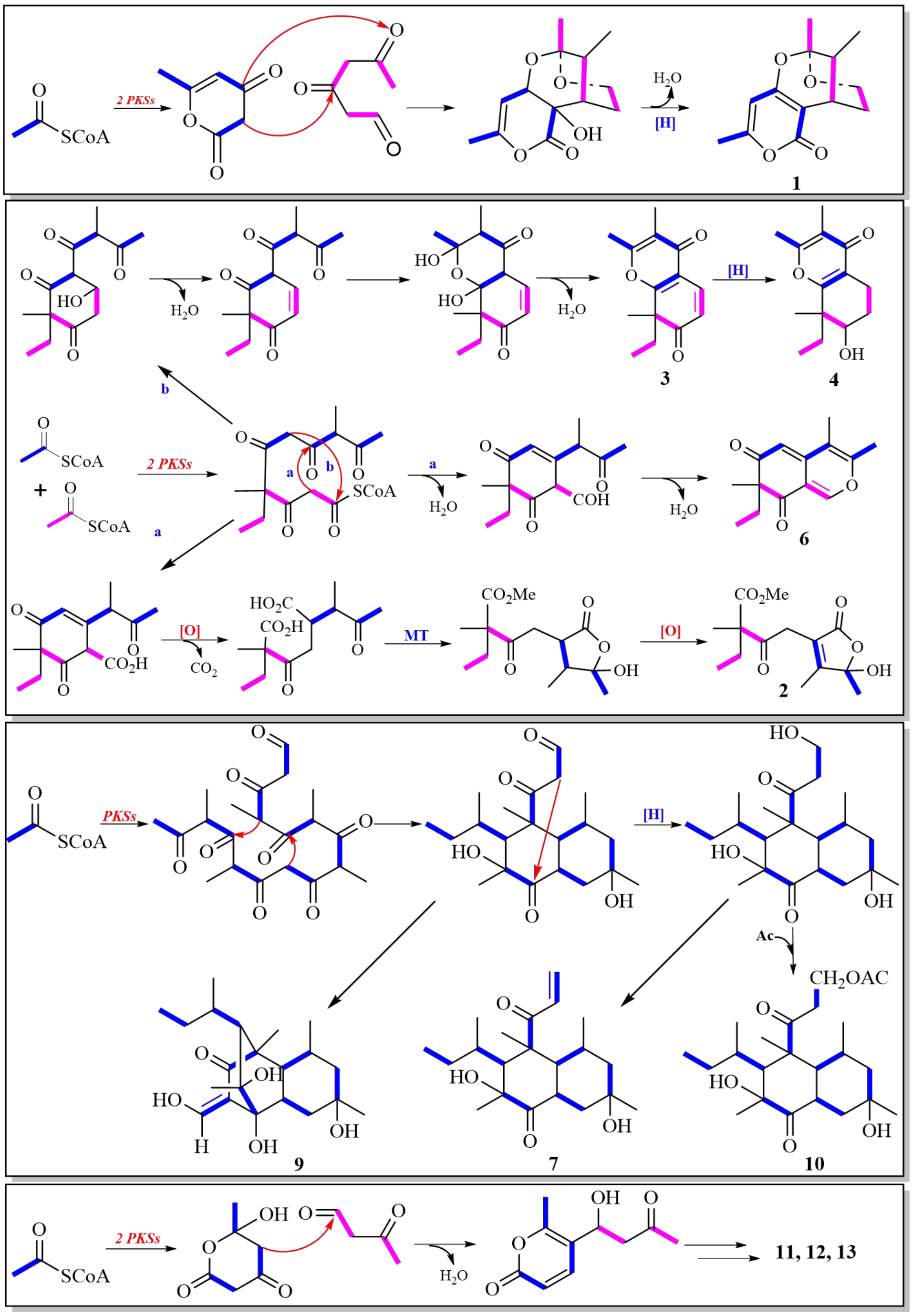

Figure S36. Phytotoxic effects on foxtail and corn leaves of **2–5**, and **7–13**.

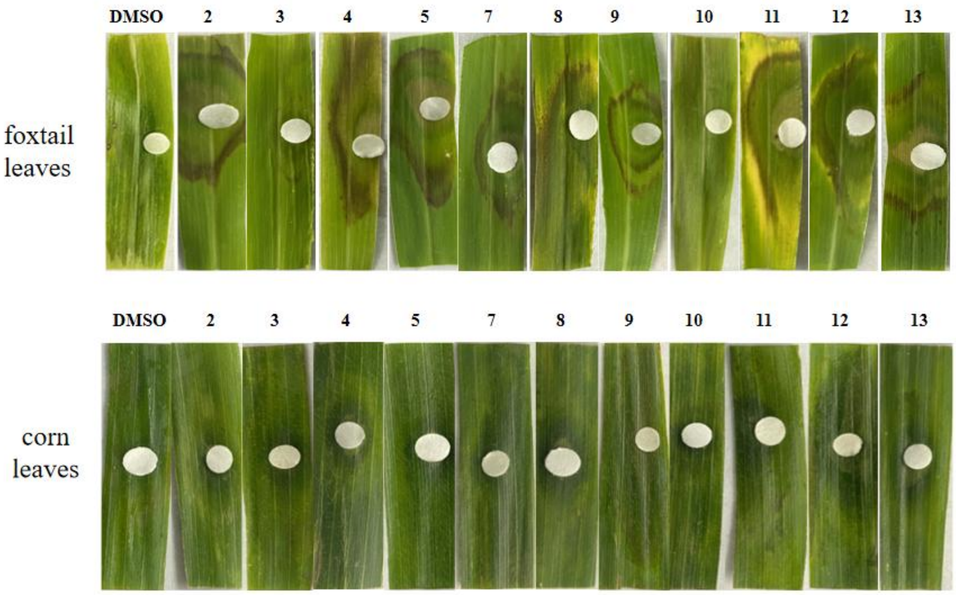

Supplement: Supplementary file 1 [file DataSheet_1.pdf]
